# The relationship between heavy metals and metabolic syndrome using machine learning

**DOI:** 10.3389/fpubh.2024.1378041

**Published:** 2024-04-15

**Authors:** Jun Yao, Zhilin Du, Fuyue Yang, Ran Duan, Tong Feng

**Affiliations:** ^1^Department of Respiratory and Critical Care, Guangyuan Central Hospital, Guangyuan, Sichuan, China; ^2^Department of Oncology, Chengdu Seventh People’s Hospital (Affliated Cancer Hospital of Chengdu Medical College), Chengdu, Sichuan, China; ^3^Department of Rheumatology and Immunology, Chengdu Fifth People's Hospital, Chengdu, Sichuan, China; ^4^Clinical Medical College, Chengdu Medical College, Chengdu, Sichuan, China; ^5^Department of Oncology, The First Aliated Hospital of Chengdu Medical College, Chengdu, Sichuan, China; ^6^The Second School of Clinical Medicine, Southern Medical University, Guangzhou, China

**Keywords:** metabolic syndrome, NHANES (National Health and Nutrition Examination Survey), machine learning, heavy metals, SHapley additive exPlanations (SHAP)

## Abstract

**Background:**

Exposure to high levels of heavy metals has been widely recognized as an important risk factor for metabolic syndrome (MetS). The main purpose of this study is to assess the associations between the level of heavy metal exposure and Mets using machine learning (ML) method.

**Methods:**

The data used in this study are from the national health and nutrition examination survey 2003–2018. According to the demographic information and heavy metal exposure level of participants, a total of 22 variables were included. Lasso was used to screen out the key variables, and 9 commonly used ML models were selected to establish the associations with the 5-fold cross validation method. Finally, we choose the SHapley Additive exPlanations (SHAP) method to explain the prediction results of Adaboost model.

**Results:**

11,667 eligible individuals were randomly divided into two groups to train and verify the prediction model. Through lasso, characteristic variables were selected from 24 variables as predictors. The AUC (area under curve) of the models selected in this study were all greater than 0.7, and AdaBoost was the best model. The AUC value of AdaBoost was 0.807, the accuracy was 0.720, and the sensitivity was 0.792. It is noteworthy that higher levels of cadmium, body mass index, cesium, being female, and increasing age were associated with an increased probability of MetS. Conversely, lower levels of cobalt and molybdenum were linked to a decrease in the estimated probability of MetS.

**Conclusion:**

Our study highlights the AdaBoost model proved to be highly effective, precise, and resilient in detecting a correlation between exposure to heavy metals and MetS. Through the use of interpretable methods, we identified cadmium, molybdenum, cobalt, cesium, uranium, and barium as prominent contributors within the predictive model.

## 1 Introduction

Metabolic syndrome (MetS) constitutes a conglomerate of disorders related to energy imbalance and metabolic dysfunction, predisposing individuals to cardiovascular diseases, diabetes, and subsequent complications, thereby elevating all-cause mortality rates and presenting a significant challenge to public health and socio-economic stability. By 2012, MetS had affected approximately one-third of the adult population in the united states (US), with a prevalence rate of 35% (1). The primary underlying mechanisms of MetS, attributed to an array of adverse lifestyle factors—including genetic predispositions, imbalanced nutritional intake, sedentarism, tobacco use, and alcohol consumption—entail the disruption of energy metabolism and the pathological accumulation of visceral fat (2–4). Moreover, environmental pollution has garnered considerable attention in recent research.

Growing epidemiological evidence suggests a link between heavy metal exposure and the risk of MetS and its components in the general population. For example, studies in Korean adults have shown that elevated blood levels of cadmium and lead correlate with a higher MetS risk, hinting at possible cumulative or synergistic effects among various heavy metals (5, 6). Nonetheless, some research has identified a negative or inverse relationship between heavy metal concentrations and MetS prevalence (7, 8). Compared to the general populace, individuals residing in heavy metal-contaminated areas—whether due to occupational or environmental factors—are at an increased risk of exposure through diverse pathways, which could affect the correlation between metal exposure and MetS risk. In Taiwan, particularly in industrial regions, there is a noted increase in MetS prevalence and blood glucose levels with rising arsenic exposure (9). Furthermore, MetS patients in areas endemic with arsenic-related diseases often report a history of consuming arsenic-laden water, and early arsenic exposure is linked to higher rates of hypertension and dyslipidemia (10, 11). While many studies have established a robust connection between chronic heavy metal exposure and Mets, the complex, nonlinear relationship between heavy metals and MetS complicates the use of traditional linear statistical methods. Moreover, epidemiological research frequently focuses on the effects of individual metals, neglecting the potential interplay among multiple metals and the common scenario of simultaneous exposure, which limits the ability to interpret complex health outcomes effectively.

To address this challenge, epidemiologists are increasingly adopting machine learning (ML) techniques known for their interpretability. Unlike traditional logistic regression, ML approaches offer several key advantages in the realms of medical research and healthcare applications (12). First, ML algorithms exhibit remarkable adaptability, adeptly managing complex, non-linear relationships among variables (13). This adaptability enables the detection of nuanced patterns and interactions within the data, enhancing prediction accuracy and overall model efficacy. Second, ML techniques generally exhibit greater resilience to outliers compared to logistic regression, managing extreme values with higher efficiency and less susceptibility to bias (14). Lastly, ML approaches enhance modeling of complex relationships in medical research and healthcare through their inherent flexibility, automation, and robustness (15).

In this study, we aimed to explore the associations between heavy metals and MetS using data from the National Health and Nutrition Examination Survey (NHANES, 2003–2018). We assessed nine ML models for their efficacy in detecting MetS from heavy metal exposure levels. Furthermore, we employed an advanced ML technique involving SHapley Additive exPlanations (SHAP) to shed light on the contribution of individual heavy metals to MetS detection. This methodology seeks to: (1) quantify the impact of each heavy metal on the ML models’ predictions; (2) explore the total effect of different heavy metals on metabolic syndrome and (3) enhance the development of early detection and intervention strategies specific to heavy metal exposure.

## 2 Materials and methods

### 2.1 Study population

The NHANES initiated in the early 1960s, is a comprehensive program aimed at evaluating the health and nutritional status of adults and children in the US. As a key initiative of the National Center for Health Statistics (NCHS), which is under the Centers for Disease Control and Prevention (CDC), NHANES uniquely integrates interviews with physical examinations to generate essential health statistics for the nation. Since 1999, NHANES has operated continuously, adapting its focus to address evolving health and nutrition issues and examining approximately 5,000 nationally representative individuals annually across various counties, with 15 counties selected each year (16).

The survey’s methodology includes both a detailed interview, covering demographic, socioeconomic, dietary, and health-related aspects, and a comprehensive examination that entails medical, dental, and physiological assessments, along with laboratory tests, conducted by skilled medical professionals. The selection process for NHANES is designed to reflect the demographic composition of the U.S. population, with particular emphasis on over-sampling older adults, African Americans, and Hispanics to ensure accurate and representative data.

Participants undergo a thorough examination by a physician, which includes dietary assessments and body measurements for all, blood sampling and dental screenings for most, and age-dependent tests and procedures. Data collection occurs in participants’ homes and in state-of-the-art mobile examination centers that are equipped to travel nationwide. The NHANES team, comprising physicians, technicians, and interviewers, utilizes advanced technology for data collection and processing, significantly reducing reliance on paper forms and manual coding.

Ethical approval for NHANES protocols was granted by the National Center for Health Statistics’ research ethics review committee, with all participants providing written informed consent.

For this analysis, we compiled NHANES data from 2003 to 2018, focusing on blood and urine metal levels and pertinent covariates, initially involving 80,312 participants. Exclusions were made for pregnant women or individuals under 20 years of age (32,723), those lacking heavy metal level data (33,120), and subjects with incomplete MetS information (3). Ultimately, 11,667 adults aged 20–79 were selected for the study. The selection process is illustrated in Supplementary Figure S1.

### 2.2 Data collection

#### 2.2.1 Participant demographic data

Basic characteristics such as age, race, gender, family poverty to income ratio (PIR), education level, smoking and alcohol consumption were obtained through questionnaire surveys (17).

#### 2.2.2 Analysis of heavy metals

In this study, 18 kinds of heavy metals in urine and blood were analyzed. All samples were collected during laboratory examination, and blood and urine samples were stored under appropriate freezing (−30 ° C) conditions until the day of detection. The whole blood and urine concentrations of heavy metals were determined by inductively coupled plasma mass spectrometry (ICP-MS) (16).

During the sample preparation phase of study, researchers subjected whole blood specimens to vortexing to achieve homogeneous distribution of cellular elements, followed by the extraction of a precise volume for metal concentration analysis. This procedure is pivotal, especially for metals predominantly located in red blood cells, such as lead, to ensure the representation of the specimen’s mean metal content. The addition of anticoagulants, notably ethylene diamine tetraacetic acid, is critical to prevent coagulation and preserve the uniformity of the sample, as coagulation can hinder the accurate sampling from the bulk specimen.

The dilution protocol preceding the analysis entails a standardized mixture of the sample with water and a specific diluent. This diluent comprises agents that liberate metals from red blood cells to facilitate ionization, mitigate ionization suppression, avert blockages due to biological matter, and incorporate internal standards to enhance analytical precision. Key diluent components, including Tetramethylammonium hydroxide and Triton X-100™, are instrumental in dissolving blood constituents and safeguarding the analytical instruments from contamination.

For the analytical phase, researchers employ an ICP-MS, which transforms liquid samples into aerosols, subsequently ionized within a plasma field, before their admission into the mass spectrometer. This stage demands meticulous temperature regulation and the application of internal standards to compensate for variations in the instrument’s performance. The spectrometer’s Dynamic Reaction Cell (DRC) is capable of operating in distinct modes, thereby amplifying specificity and sensitivity by diminishing interference, a crucial feature for analyzing elements like manganese, mercury, and selenium.

When the concentration of biomarkers is lower than the detection limit, the limit is divided by the square root of 2 according to NHANES scheme. See NHANES website for detailed determination methods. The NHANES quality assurance and quality control protocol meets the requirements of the clinical laboratory improvement act of 1988.

#### 2.2.3 Ascertainment of outcomes

Metabolic syndrome can be diagnosed if the following ≥3 items are met (18):

1. Hypertension is systolic blood pressure ≥ 130 mmHg, diastolic blood pressure ≥ 85 mmHg, or has been diagnosed with hypertension and treated;
2. Fasting triglyceride ≥150 mg/dL, or the current use of drugs to treat high triglyceride;
3. Female HDL-c < 50 mg/dL, male hdl-c < 40 mg/dL, or the current use of drugs to reduce HDL;
4. Female waist circumference ≥ 88 cm, male waist circumference ≥ 102 cm;
5. Hyperglycemia is defined as fasting blood glucose ≥100 mg/dL or diabetes mellitus diagnosed and treated.

### 2.3 Data preprocessing and feature filtering

The data set was initially composed of 24 variables, called features in ML. It can be seen from Supplementary Tables S1, S2 that most of the data in this study sample are complete, and the missing data are less than 10%. According to the type of missing values, random forest filling method is used to deal with the missing data and the abnormal value is handled (Supplementary Tables S3, S4). The distribution of interpolated data is similar to the observed data (Supplementary Figure S2). In order to ensure that the data follow the normal distribution in the subsequent analysis, we performed logarithmic transformation on metal variables (Supplementary Table S5). Collinearity makes the parameter estimation of the model inaccurate, resulting in the model being too complex and over fitting the training data. We performed Pearson correlation analysis to test the relationship between these metals (Supplementary Figure S3). Using the least absolute shrinkage and selection operator (LASSO), the regularization term is introduced into the loss function to punish the model parameters and reduce the influence of collinearity. Variance inflation factor (VIF) is used to help identify the high correlation between independent variables, where VIF value below 10 indicates that there is no multicollinearity.

### 2.4 ML model strategies

The purpose of the multi-model classification approach is to identify the optimal model type, rather than to directly construct the final model. It employs a resampling mechanism (similar to k-fold) for training/validation to deduce the performance of each model (e.g., average AUC scores and variance) across multiple training sessions, focusing on the overall performance of each model category within the dataset. Then we will employ the best machine learning method for classification, with a total dataset sample size of *N* = 11,667, containing the following class information: Class (0): *N* = 7,827, Class (1): *N* = 3,840. From the total sample, a test set of *N* = 2,333 (20.00%) is randomly drawn, with the remaining samples used as a training set for 5-fold cross-validation. In this study, we used nine ML algorithms, namely extreme gradient boosting (xgboost) algorithm, logistic regression (LR), random forest (RF), AdaBoost, gaussian Nb (GNB), complementnb (CNB), multi-layer perceptron (MLP), and support vector machine (SVM) and k-nearest neighbor machine (KNN) learning models. See Supplementary Table S6 for specific parameters of each model. In order to further evaluate the predictive ability of the model for the final treatment results, the model was evaluated by area under the curve (AUC) value, accuracy, kappa coefficient, sensitivity, specificity, accuracy, recall rate, F1 and other indicators of the test data set (19).

SHAP additivity analysis is a method to explain individual prediction. It was originally evolved from the best Shapley value in game theory. The goal is to explain the prediction of instance X by calculating the contribution of each feature to prediction X. In this study, the algorithm is mainly used to sort according to the importance of variable characteristics in the model established by xgboost classifier, and screen out the top ten predictive factors in tb-dm patients. SHAP can reasonably explain the contribution value of each variable to the model, and avoid the long-standing “black box” theory in ML. Therefore, clinicians can make more optimal judgments when formulating treatment plans for patients (20).

### 2.5 Statistical analysis

**Figure 1:**
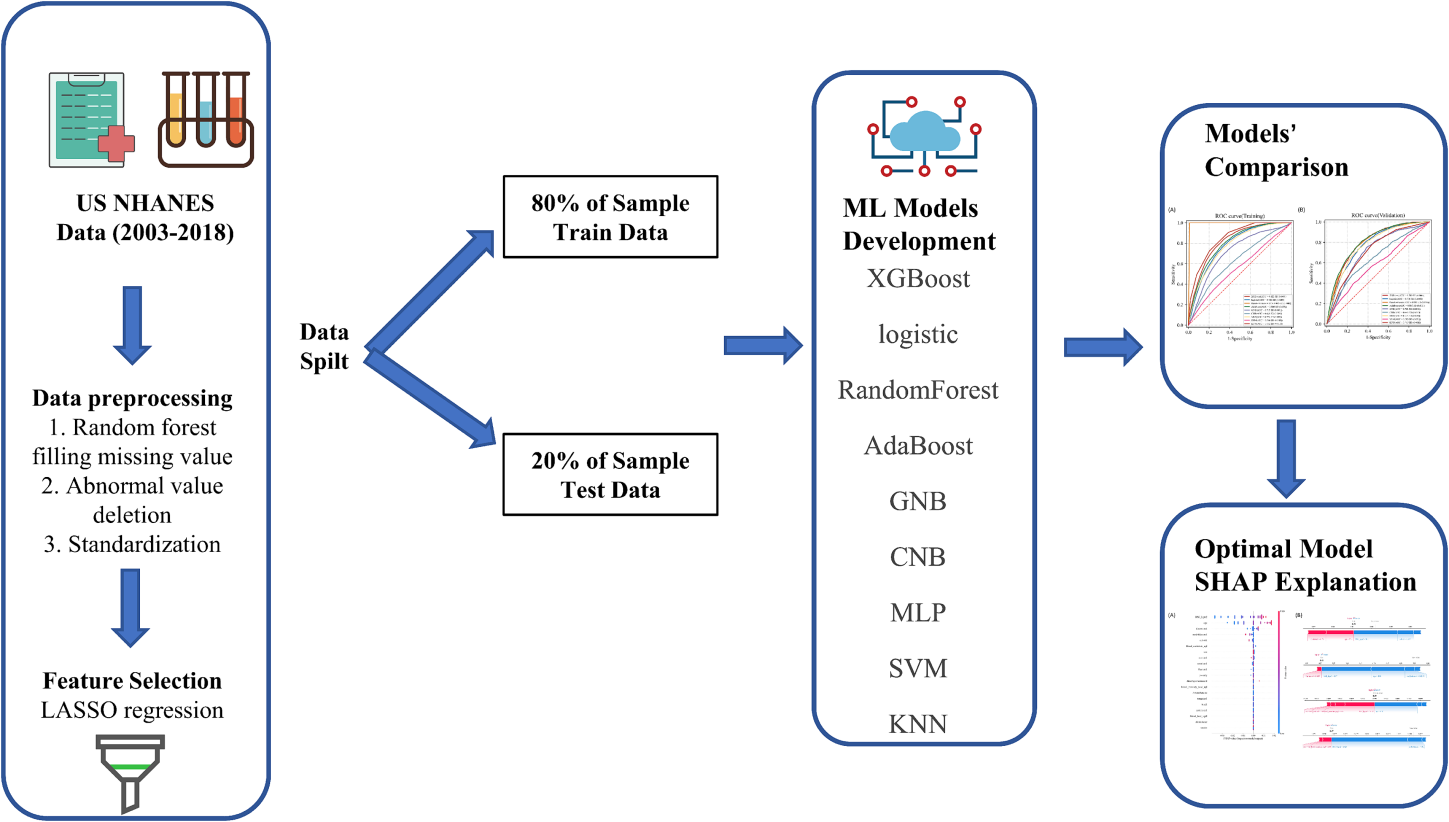
Model-making process and article framework. This figure shows how the data were obtained from electronic medical record systems, and the collection of data on all study variables, including demographic characteristics, laboratory indicators. Data on a total of 24 variables were collected, 22 of which were selected. The 22 variables were used to establish the machine learning models.

In this study, continuous variables were expressed as mean ± standard deviation or interquartile interval (IQR; 25–75%). T test was used for normal continuous variables, while Mann Whitney U test was used for non-normal continuous variables. Categorical variables are described as percentages (%). Chi square or Fisher exact probability test is used for constituent ratio comparison. All statistical analyses were conducted using Python (version 3.10.9) and R software (version 4.2.3), with the Lasso R package glmnet at version 4.1.8, XGBoost in Python at xgboost = 2.0.1, and other methods in Python using scikit-learn = 1.1.3.The overall design of the paper is shown in Figure 1.

## 3 Results

### 3.1 Baseline data comparison

Table 1 presents the demographic characteristics of the study participants. A total of 11,667 individuals were included in the analysis, with 48.99% being male and an average age of 48.0 (interquartile range, 34.0–64.0). Out of the participants, 3,840 were diagnosed with MetS. Individuals with MetS tended to be older, have a higher body weight, be non-Hispanic white, and have a higher average family income (all *p* < 0.05). The content distribution of heavy metals in blood or urine in each two cycles from 2003 to 2018 is shown in Table 2.

**Table 1:** Characteristics of the study participants with and without MetS form 2003–2018 in US NHANES.

Characteristics	Total (*n* = 11,667)	Without MetS (*n* = 7,827)	With MetS (*n* = 3,840)	*p*
Education Level, *n* (%)				<0.001
Less than high school	1,410 (12.085)	839 (10.719)	571 (14.870)	
High school or equivalent	4,359 (37.362)	2,865 (36.604)	1,494 (38.906)	
College or above	5,898 (50.553)	4,123 (52.677)	1775 (46.224)	
Alcohol user, *n* (%)				<0.001
No	1,634 (14.005)	1,052 (13.441)	582 (15.156)	
Former	2,237 (19.174)	1,358 (17.350)	879 (22.891)	
Mild	3,834 (32.862)	2,513 (32.107)	1,321 (34.401)	
Moderate	1756 (15.051)	1,286 (16.430)	470 (12.240)	
Heavy	2,206 (18.908)	1,618 (20.672)	588 (15.313)	
Smoke, *n* (%)				<0.001
No	6,367 (54.573)	4,415 (56.407)	1952 (50.833)	
Former	2,908 (24.925)	1,655 (21.145)	1,253 (32.630)	
Current	2,392 (20.502)	1757 (22.448)	635 (16.536)	
Race, *n* (%)				<0.001
Non-Hispanic Black	2,402 (20.588)	1,559 (19.918)	843 (21.953)	
Other Hispanic	1,039 (8.905)	702 (8.969)	337 (8.776)	
Non-Hispanic White	5,188 (44.467)	3,403 (43.478)	1785 (46.484)	
Mexican American	1957 (16.774)	1,319 (16.852)	638 (16.615)	
Other race – including multi-racial	1,081 (9.265)	844 (10.783)	237 (6.172)	
Sex, *n* (%)				0.078
Male	5,716 (48.993)	3,790 (48.422)	1926 (50.156)	
Female	5,951 (51.007)	4,037 (51.578)	1914 (49.844)	
Age	48.000[34.000,64.000]	42.000[30.000,58.000]	61.000 [48.000,71.000]	<0.001
Poverty	2.110 [1.130,3.920]	2.100 [1.100,3.930]	2.131 [1.180,3.900]	0.049
BMI	27.810 [24.290,32.290]	26.410 [23.250,30.620]	30.440 [27.290,34.600]	<0.001

**Table 2:** Geometric means and geometric standard deviations of heavy metals by each cycle of US NHANES (2003–2018).

Heavy metal	Year 2003–2004 (*n* = 1,488)	Year 2005–2006 (*n* = 1,457)	Year 2007–2008 (*n* = 1714)	Year 2009–2010 (*n* = 1934)	Year 2011–2012 (*n* = 1,622)	Year 2013–2014 (*n* = 1741)	Year 2015–2016 (*n* = 1711)	*p*
Blood cadmium	0.40[0.20,0.60]	0.34[0.21,0.60]	0.35 [0.23,0.62]	0.35 [0.22,0.62]	0.33 [0.21,0.61]	0.30 [0.18,0.58]	0.30 [0.18,0.53]	<0.001
Blood lead	1.70 [1.10,2.60]	1.48 [0.92,2.37]	1.41 [0.96,2.24]	1.29 [0.84,2.00]	1.08 [0.72,1.71]	1.00 [0.64,1.56]	0.97 [0.60,1.59]	<0.001
Blood mercury total	0.90 [0.50,1.70]	0.94 [0.50,1.73]	0.87 [0.48,1.58]	0.93 [0.50,1.85]	0.88 [0.44,1.88]	0.79 [0.42,1.63]	0.77 [0.42,1.55]	<0.001
Arsenous acid	0.80 [0.80,0.80]	0.85 [0.85,0.85]	0.85 [0.85,0.85]	0.85 [0.85,0.85]	0.34 [0.34,0.56]	0.46 [0.08,0.73]	0.14 [0.08,0.56]	<0.001
Arsenic acid	0.70 [0.70,0.70]	0.71 [0.71,0.71]	0.71 [0.71,0.71]	0.71 [0.71,0.71]	0.62 [0.62,0.62]	0.56 [0.56,0.56]	0.56 [0.56,0.56]	<0.001
Arsenobetaine	1.40 [0.30,6.10]	1.85 [0.40,7.42]	0.98 [0.28,5.07]	1.27 [0.28,7.02]	1.40 [0.84,7.39]	1.17 [0.82,4.85]	0.82 [0.82,5.64]	<0.001
Arsenocholine	0.40 [0.40,0.40]	0.42 [0.42,0.42]	0.42 [0.42,0.42]	0.42 [0.42,0.42]	0.20 [0.20,0.20]	0.08 [0.08,0.08]	0.08 [0.08,0.08]	<0.001
Dimethylarsonic acid	4.00 [2.00,6.00]	3.96 [2.39,6.46]	3.70 [2.28,6.42]	3.69 [2.06,6.59]	3.99 [2.16,7.44]	3.33 [1.35,5.66]	3.35 [1.35,5.87]	<0.001
Barium	1.35 [0.66,2.44]	1.31 [0.68,2.57]	1.32 [0.66,2.46]	1.30 [0.66,2.48]	1.08 [0.54,2.17]	0.93 [0.48,1.93]	1.03 [0.51,2.03]	<0.001
Cadmium	0.30 [0.15,0.56]	0.27 [0.13,0.52]	0.27 [0.14,0.52]	0.26 [0.13,0.48]	0.22 [0.11,0.44]	0.18 [0.08,0.38]	0.20 [0.09,0.42]	<0.001
Cobalt	0.31 [0.19,0.49]	0.36 [0.23,0.58]	0.34 [0.21,0.53]	0.34 [0.20,0.54]	0.30 [0.18,0.48]	0.38 [0.22,0.64]	0.40 [0.24,0.63]	<0.001
Cesium	4.84 [2.84,7.30]	5.06 [3.07,7.59]	4.72 [2.95,6.92]	4.37 [2.76,6.50]	4.19 [2.56,6.36]	4.09 [2.47,6.46]	4.45 [2.68,6.53]	<0.001
Lead	0.70 [0.40,1.17]	0.67 [0.37,1.15]	0.59 [0.32,0.97]	0.53 [0.31,0.90]	0.41 [0.24,0.73]	0.33 [0.18,0.57]	0.35 [0.19,0.62]	<0.001
Antimony	0.07 [0.05,0.12]	0.07 [0.04,0.12]	0.05 [0.03,0.09]	0.05 [0.02,0.09]	0.04 [0.02,0.07]	0.04 [0.01,0.07]	0.04 [0.02,0.08]	<0.001
Thallium	0.16 [0.09,0.25]	0.16 [0.10,0.25]	0.14 [0.09,0.23]	0.15 [0.08,0.23]	0.16 [0.09,0.25]	0.15 [0.08,0.23]	0.16 [0.09,0.25]	<0.001
Tungsten	0.06 [0.03,0.11]	0.07 [0.03,0.14]	0.08 [0.04,0.17]	0.07 [0.03,0.13]	0.06 [0.03,0.13]	0.05 [0.02,0.11]	0.05 [0.02,0.11]	<0.001
Uranium	0.007 [0.004,0.012]	0.005 [0.003,0.010]	0.007 [0.004,0.013]	0.007 [0.004,0.014]	0.005 [0.002,0.011]	0.005 [0.002,0.011]	0.005 [0.003,0.010]	<0.001
Molybdenum	41.50 [22.60,71.0]	46.70 [25.70,75.10]	45.60 [23.80,79.10]	42.20 [23.90,73.50]	40.20 [21.30,67.50]	34.46 [17.72,62.15]	38.50[19.44,66.70]	<0.001

### 3.2 Feature selection

**Figure 2:**
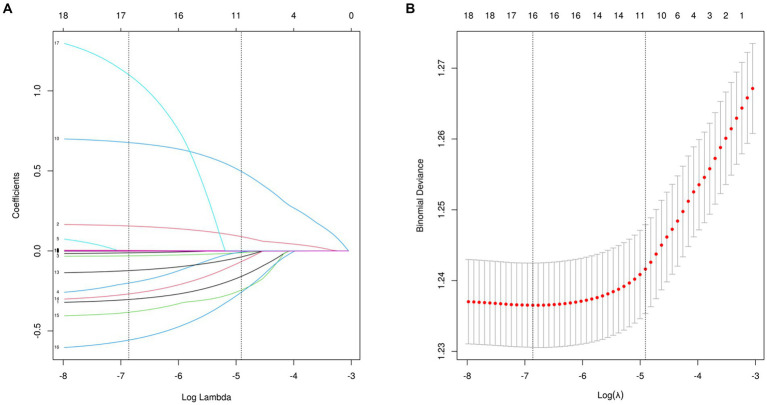
Features selection used the LASSO regression. **(A)** A coefficient profile plot is created against the logarithmic (lambda) sequence. In this study, the selection of predictors is based on the minimum criteria (indicated by the left dotted line), resulting in the selection of 16 nonzero coefficients using the LASSO regression model. **(B)** The tuning parameter (lambda) selection is based on the deviance in the LASSO regression, using both the minimum criteria (indicated by the left dotted line) and the 1-SE criteria (indicated by the right dotted line). LASSO, least absolute shrinkage and selection operator; SE, standard error.

16 variables were selected using LASSO regression analysis based on their non-zero coefficients, as shown in Figure 2. These selected variables were blood cadmium, blood lead, blood mercury total, arsenous acid, arsenobetaine, arsenocholine, dimethylarsonic acid, barium, cadmium, cesium, lead, antimony, thallium, tungsten, uranium, molybdenum. The evaluation of multicollinearity among the various chosen metals and covariates using VIFs revealed no evidence of multicollinearity (Supplementary Table S7).

### 3.3 Evaluation and comparison of the model

**Figure 3:**
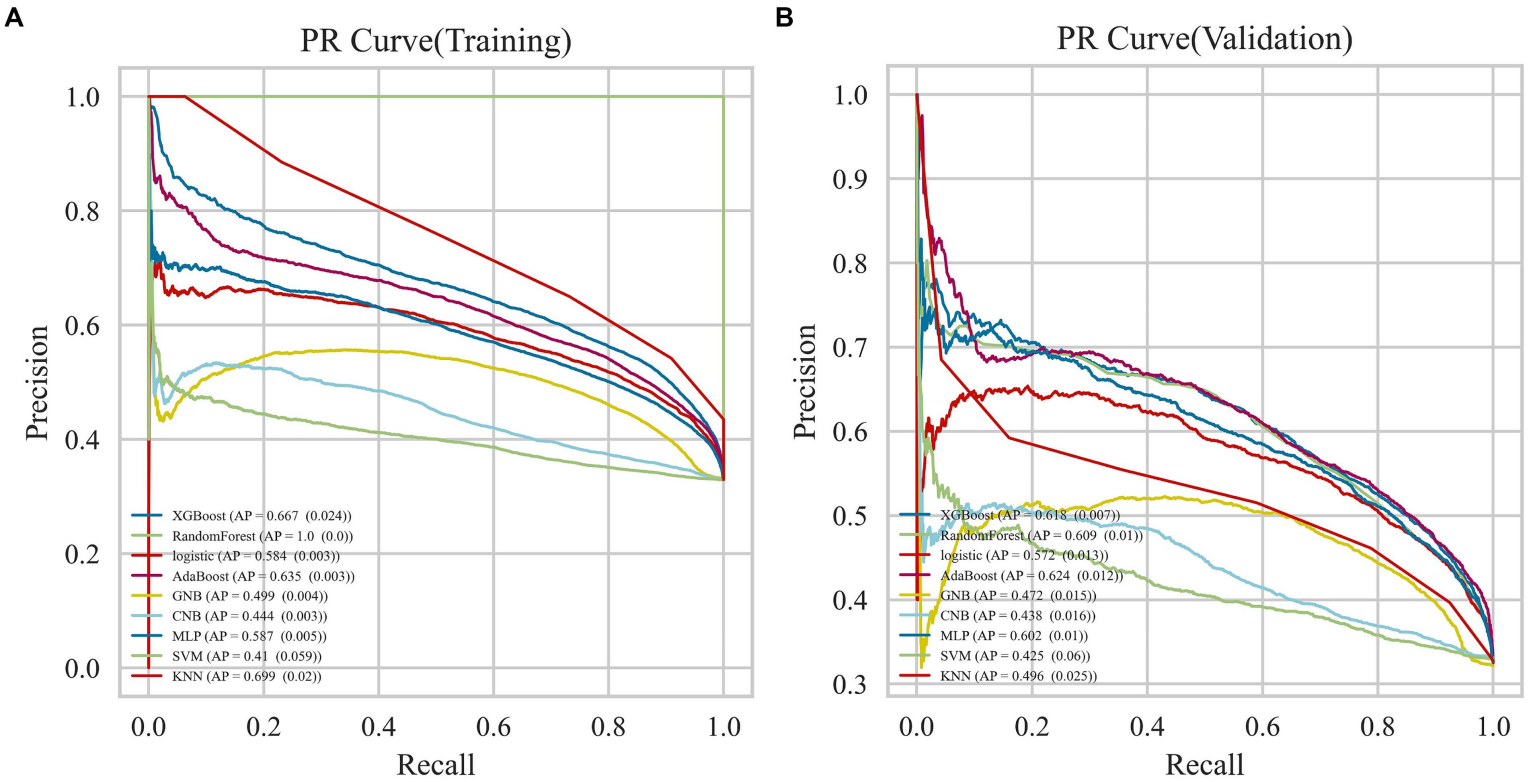
AUC curves for nine machine learning models. The AdaBoost model achieved a larger (better) AUC compared with the other models. **(A)** Training dataset **(B)** Validation dataset.

**Figure 4:**
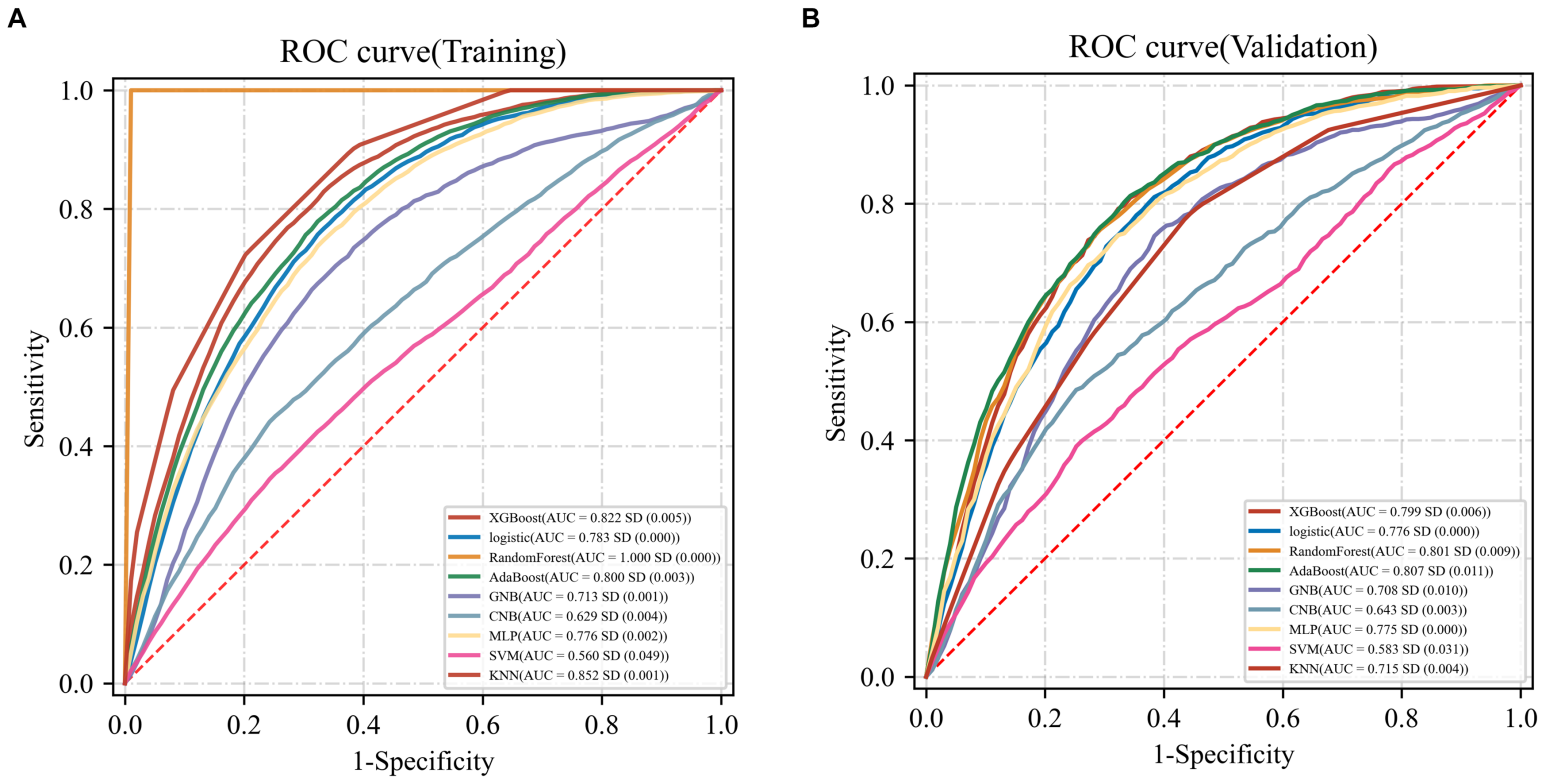
Precision-recall curve curves for nine machine learning models. **(A)** Training dataset **(B)** Validation dataset.

**Figure 5:**
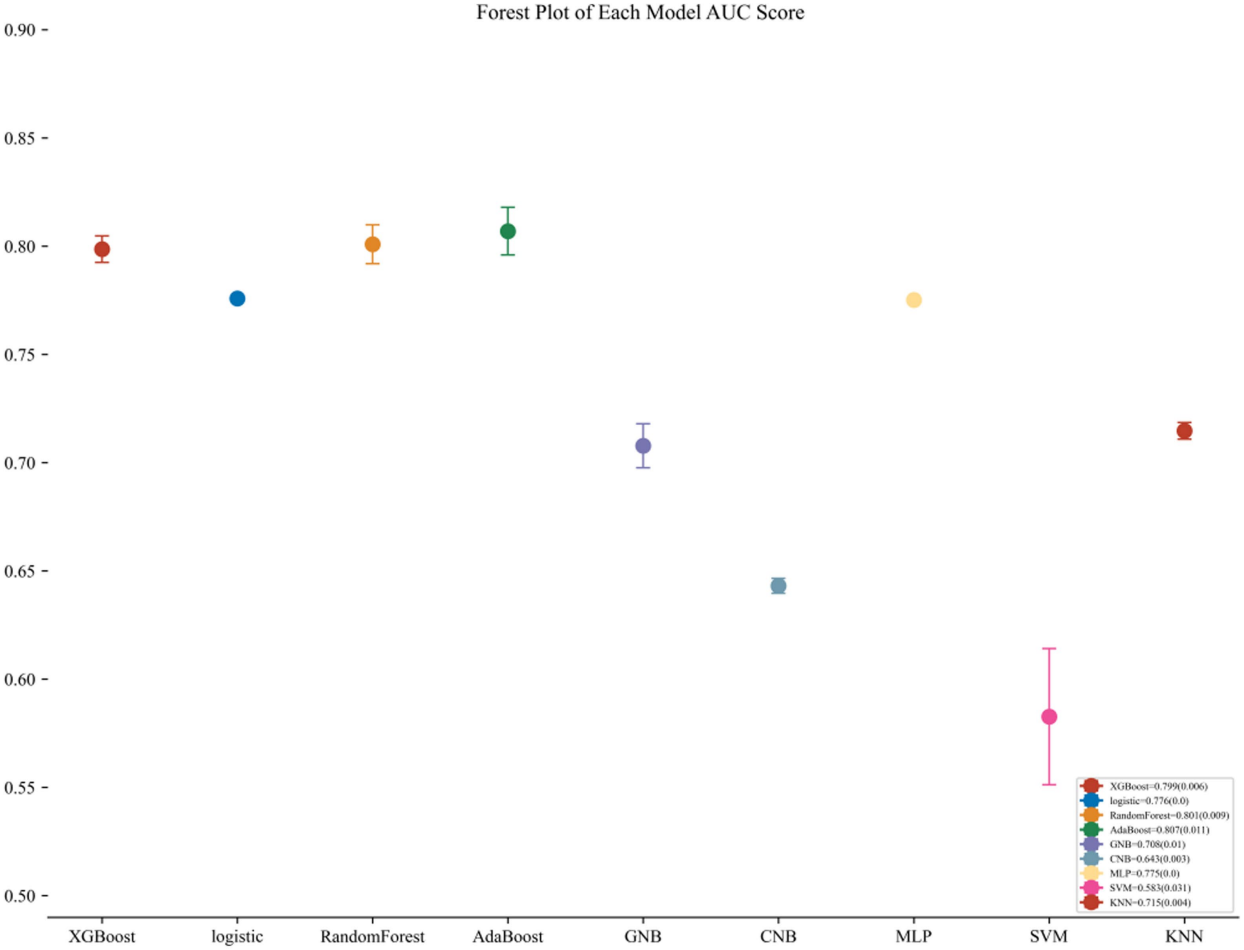
Forest plot of the AUC Score of the nine models. The AdaBoost model achieved a smaller (better) standard deviation (SD) compared with the other models.

**Figure 6:**
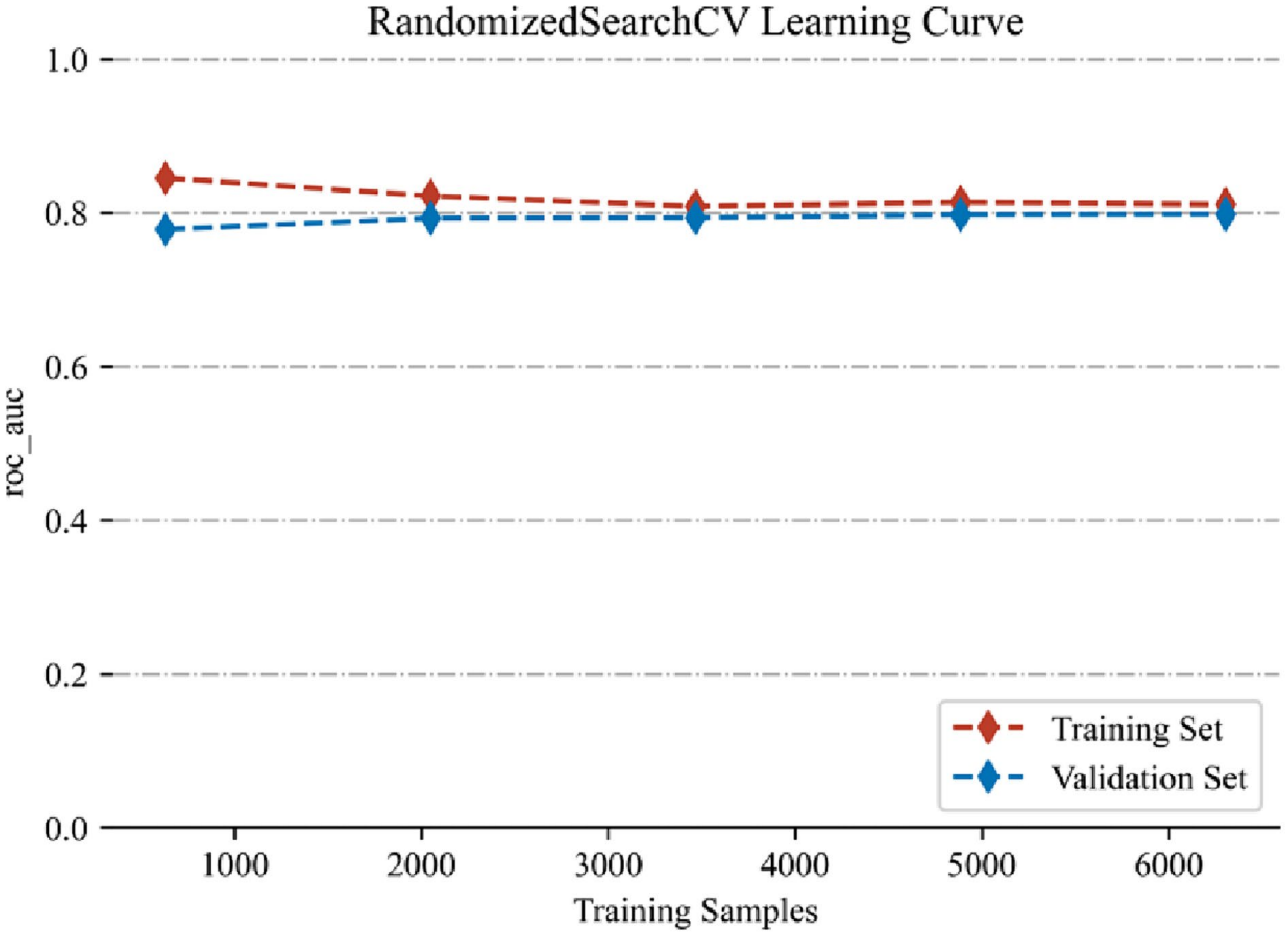
AUC performance of training set and validation set.

The AdaBoost model exhibited superior performance compared to other models, achieving a larger AUC as depicted in Figure 3 and Supplementary Table S8. Precision-recall curve are shown in Figure 4. A forest plot of the AUC score for the multiple models based on the AUC of the nine models was created (Figure 5), with the AdaBoost algorithm demonstrating the best predictive performance, achieving an AUC of 0.807. Since the performance of the validation set under the AUC index does not exceed the test set or the exceeding ratio is less than 10%, it can be considered that the fitting is successful (Figure 6). Consequently, the AdaBoost algorithm was chosen for further analyses. The training dataset values are presented in Table 3, and the validation set values are available in Table 4. Supplementary Figure S4 displays the confusion matrix for the nine ML algorithms used.

**Table 3:** Performance metrics for nine models in the training dataset.

Characteristics	AUC	Cutoff	Accuracy	Sensitivity/Recall	Specificity	PPV	NPV	F1 score	Kappa
XGB	0.822 (0.005)	0.357 (0.038)	0.730 (0.011)	0.808 (0.040)	0.690 (0.037)	0.571 (0.016)	0.877 (0.017)	0.668 (0.003)	0.452 (0.008)
logistic	0.783 (0.000)	0.313 (0.009)	0.698 (0.006)	0.777 (0.014)	0.659 (0.016)	0.533 (0.005)	0.855 (0.003)	0.632 (0.001)	0.390 (0.005)
RF	1.000 (0.000)	0.535 (0.015)	1.000 (0.000)	1.000 (0.000)	1.000 (0.000)	1.000 (0.000)	1.000 (0.000)	1.000 (0.000)	1.000 (0.000)
AB	0.800 (0.003)	0.475 (0.012)	0.711 (0.001)	0.783 (0.003)	0.674 (0.001)	0.552 (0.000)	0.858 (0.003)	0.647 (0.001)	0.414 (0.002)
GNB	0.713 (0.001)	0.923 (0.023)	0.664 (0.007)	0.715 (0.022)	0.638 (0.021)	0.500 (0.010)	0.815 (0.009)	0.589 (0.001)	0.319 (0.004)
CNB	0.629 (0.004)	0.986 (0.006)	0.649 (0.002)	0.453 (0.006)	0.748 (0.008)	0.476 (0.008)	0.730 (0.003)	0.464 (0.001)	0.203 (0.004)
MLP	0.776 (0.002)	0.329 (0.023)	0.692 (0.005)	0.757 (0.013)	0.660 (0.014)	0.530 (0.007)	0.843 (0.005)	0.623 (0.001)	0.377 (0.005)
SVM	0.560 (0.049)	0.437 (0.039)	0.536 (0.111)	0.627 (0.178)	0.488 (0.260)	0.413 (0.063)	0.710 (0.017)	0.475 (0.013)	0.113 (0.088)
KNN	0.852 (0.001)	0.500 (0.000)	0.776 (0.001)	0.723 (0.002)	0.798 (0.001)	0.758 (0.001)	0.781 (0.001)	0.740 (0.001)	0.451 (0.004)

XGB, extreme gradient boosting; RF, random forest; AB, AdaBoost; SVC, support vector classification; NB, Naive Bayes; MLP, multilayer perceptron; SVM, support vector machine; KNN, K-Nearest Neighbor; AUC, area under the receiver operator curve; FPR, false positive rate; FNR, false negative rate; PPV, positive predictive value; NPV, negative predictive value.

**Table 4:** Performance metrics for nine models in the validation dataset.

Characteristics	AUC	Cutoff	Accuracy	Sensitivity/Recall	Specificity	PPV	NPV	F1 score	Kappa
XGB	0.799 (0.006)	0.357 (0.038)	0.713 (0.010)	0.778 (0.034)	0.694 (0.034)	0.555 (0.017)	0.863 (0.010)	0.647 (0.000)	0.420 (0.012)
logistic	0.776 (0.000)	0.313 (0.009)	0.694 (0.005)	0.773 (0.039)	0.658 (0.033)	0.548 (0.015)	0.841 (0.015)	0.640 (0.003)	0.388 (0.004)
RF	0.801 (0.009)	0.535 (0.015)	0.736 (0.003)	0.758 (0.005)	0.706 (0.012)	0.667 (0.018)	0.754 (0.004)	0.710 (0.012)	0.339 (0.012)
AB	0.807 (0.011)	0.475 (0.012)	0.720 (0.009)	0.792 (0.016)	0.686 (0.031)	0.556 (0.016)	0.862 (0.001)	0.653 (0.005)	0.426 (0.017)
GNB	0.708 (0.010)	0.923 (0.023)	0.658 (0.006)	0.755 (0.015)	0.609 (0.004)	0.500 (0.017)	0.815 (0.006)	0.602 (0.017)	0.315 (0.020)
CNB	0.643 (0.003)	0.986 (0.006)	0.658 (0.006)	0.488 (0.006)	0.746 (0.010)	0.508 (0.011)	0.730 (0.012)	0.498 (0.008)	0.234 (0.002)
MLP	0.775 (0.000)	0.329 (0.023)	0.697 (0.006)	0.697 (0.025)	0.733 (0.023)	0.545 (0.004)	0.835 (0.004)	0.611 (0.007)	0.385 (0.003)
SVM	0.583 (0.031)	0.437 (0.039)	0.539 (0.105)	0.649 (0.206)	0.495 (0.256)	0.412 (0.063)	0.744 (0.026)	0.477 (0.019)	0.125 (0.062)
KNN	0.715 (0.004)	0.500 (0.000)	0.692 (0.009)	0.790 (0.002)	0.551 (0.011)	0.578 (0.035)	0.722 (0.003)	0.667 (0.024)	0.245 (0.029)

XGB, extreme gradient boosting; RF, random forest; AB, AdaBoost; SVC, support vector classification; NB, Naive Bayes; MLP, multilayer perceptron; SVM, support vector machine; KNN, K-Nearest Neighbor; AUC, area under the receiver operator curve; FPR, false positive rate; FNR, false negative rate; PPV, positive predictive value; NPV, negative predictive value.

### 3.4 Visualization of feature importance

**Figure 7:**
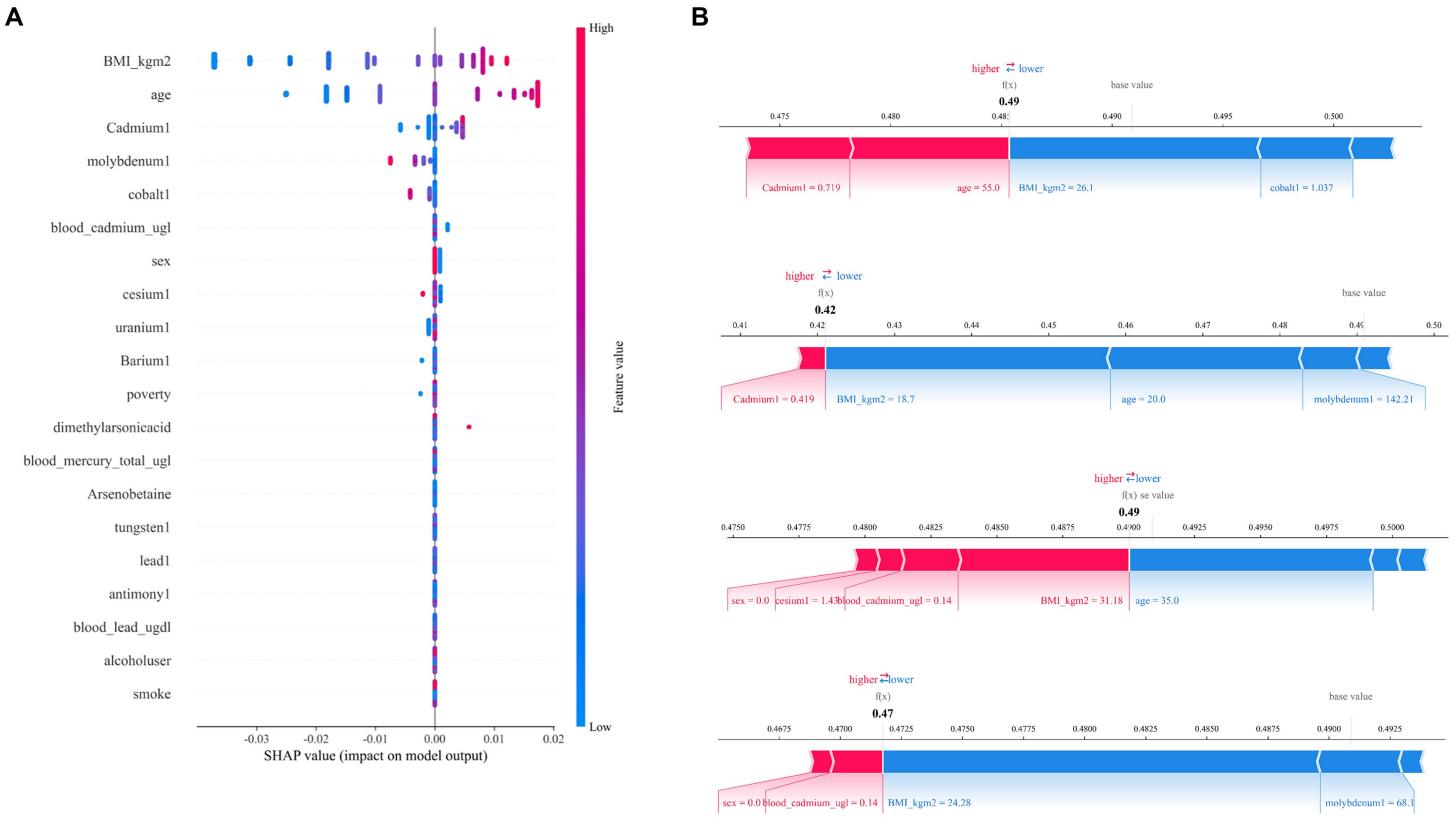
SHAP analysis of the AdaBoost model. **(A)** A visual representation of each feature of the AdaBoost model, showing the relationship between the importance of each feature. The color represents the value of the variable, with red representing the larger value and blue representing the smaller value. **(B)** The contributing variables are arranged in the horizontal line, sorted by the absolute value of their impact.

**Figure 8:**
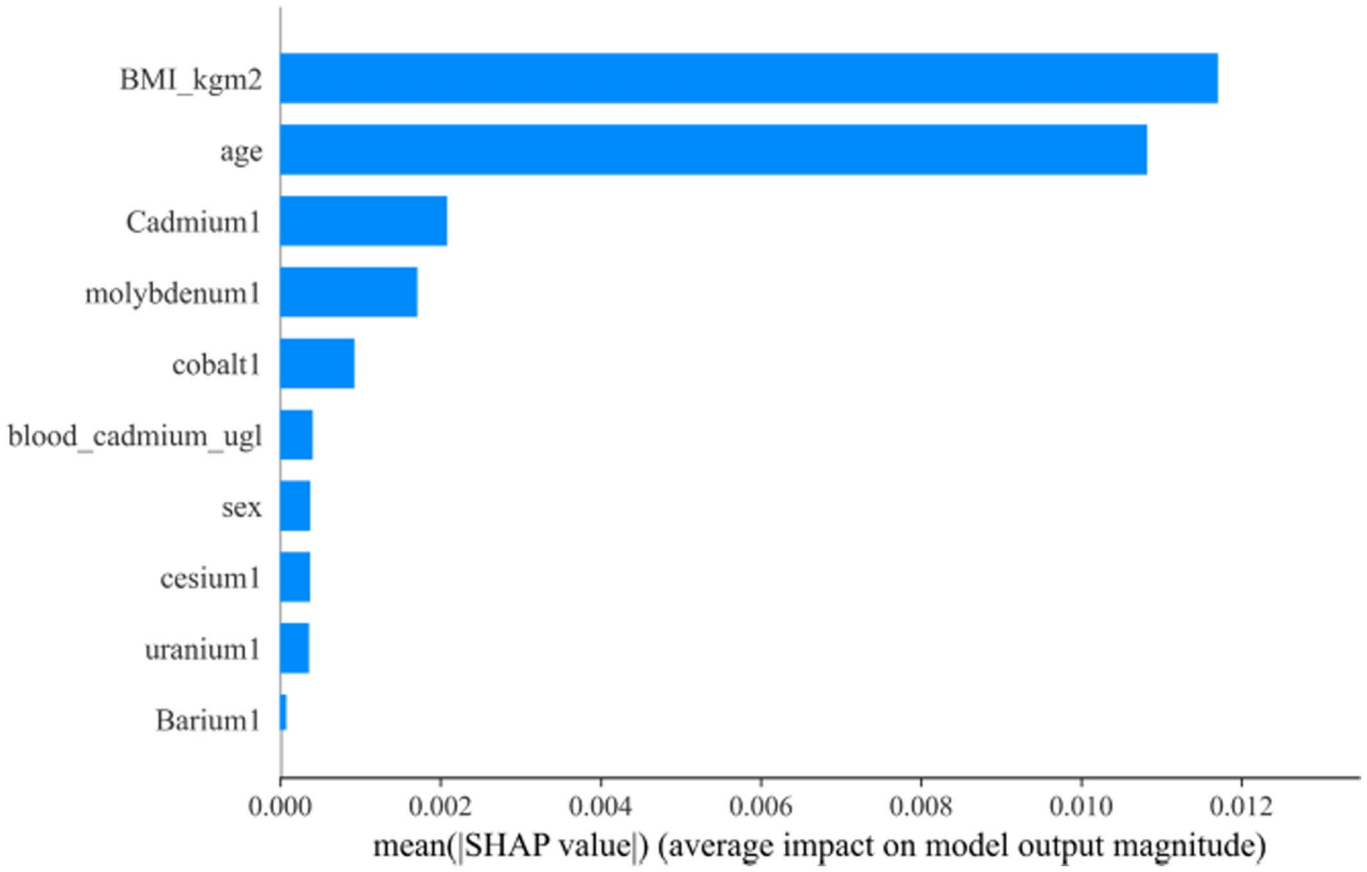
“SHAP” package to explain the importance of key variables to the model.

After the above analysis, we use the SHAP method to explain the model established by AdaBoost. Figures 7, 8 shows the contribution of each screened feature to the model obtained by the SHAP method. Figure 8 offers an illustration of the assessment of MetS risk, showing the influence of features such as cadmium, body mass index (BMI), cesium, gender, and age on the estimated probability of MetS.

## 4 Discussion

In our study, we utilized a ML approach, complemented by an intuitive process, to investigate the relationships between heavy metal exposure levels and Mets. We developed nine ML models to detect MetS, achieving noteworthy predictive accuracy and interpretability from heavy metal datasets. The AdaBoost model, in particular, exhibited outstanding performance, pinpointing cadmium, molybdenum, cobalt, cesium, uranium, and barium as critical contributors to MetS detection. This ML model holds promise for supporting the creation of tailored healthcare strategies for individuals, based on their specific heavy metal exposure profiles.

This study extends previous work that employed ML algorithms for disease prediction, as demonstrated in several key studies (21–23). These investigations have underscored the capability of sophisticated classification algorithms to enhance prediction accuracy. In recent years, ML algorithms have increasingly contributed valuable insights to clinical decision-making processes. Leveraging vast clinical datasets, ML has rapidly advanced and proven its efficacy in forecasting the outcomes of various diseases (14). ML algorithms excel at synthesizing and analyzing large volumes of diverse data, a task that poses significant challenges for human analysts. Nonetheless, interpreting the outputs of ML algorithms remains complex (24). To address this complexity, we utilized SHAP values within the AdaBoost model to facilitate optimal interpretation and elucidate their influence on the predictive outcomes. In the context of the 2003–2018 NHANES survey, a positive SHAP value suggests that certain features elevate MetS risk, whereas a negative SHAP value indicates a lower risk.

This research revealed significant correlations between MetS and exposure to certain heavy metals, emphasizing the importance of recognizing the various dietary pathways through which these metals can be ingested. For instance, cadmium, commonly found in cereals, leafy vegetables, and shellfish, and chromium, present in meat, whole grains, and fruits, represent notable sources of exposure (25). Additionally, the consumption of fatty fish, dairy products, and meats may lead to exposure to Persistent Organic Pollutants (POPs), such as polychlorinated biphenyls (PCBs) and dioxins, which accumulate in the food chain and are associated with various negative health outcomes, including metabolic disorders (26–28). The identification of these heavy metals as factors contributing to MetS highlights the urgent need for comprehensive public health measures to mitigate exposure to these detrimental elements. Such measures could encompass policy-level initiatives to enforce stricter controls on industrial discharges and agricultural chemicals, alongside community and individual initiatives to enhance awareness of heavy metal and POP sources. Public health campaigns and dietary guidelines could also be instrumental in reducing consumption of contaminated foods. Furthermore, healthcare professionals have a pivotal role in screening for heavy metal exposure among susceptible groups and advising on dietary modifications to reduce such exposure.

The findings derived from SHAP analysis align with prior research outcomes. Specifically, Lee and Kim identified a notable positive association between blood cadmium levels and MetS risk in Korean males, utilizing data from the Korean National Health and Nutrition Examination Survey for the periods 2005–2010 and 2008–2012 (5, 29). Similarly, an Iranian study reported elevated urine cadmium levels in individuals with MetS (30). Additionally, experimental evidence revealed that serum chromium concentrations were lower in the diabetes group (0.0205 ± 0.0012 μg/mg) compared to the control group (0.0267 ± 0.0009 μg/mg). This finding was corroborated by Flores et al., who reported serum chromium levels in healthy individuals and diabetes patients to be 1.44 μg/L and 0.66 μg/L, respectively, further substantiating the observed disparity (31). From a mechanistic perspective, chromium enhances insulin sensitivity by activating insulin receptor kinase and facilitating insulin’s interaction with its cellular receptors, thus amplifying its biological efficacy.

Metals exhibit either additive or synergistic effects due to shared exposure routes and mechanisms of action, with oxidative stress being a principal shared pathway (32). Chronic exposure to heavy metals such as cadmium and lead results in glutathione depletion and the binding to sulfhydryl groups in proteins (33). The oxidation of Arsenite (As III) to Arsenate (As V) leads to the formation of hydrogen peroxide and the interaction with critical thiol groups (34). This cascade triggers an extensive production of free radicals, reactive oxygen species (ROS), and reactive nitrogen species (RNS), disrupting the balance within the antioxidant/oxidant system. The ensuing oxidative stress culminates in lipid peroxidation, Deoxyribo Nucleic Acid (DNA) and cellular membrane damage, protein alterations, and other detrimental effects, ultimately contributing to the onset of chronic conditions such as diabetes and cardiovascular diseases (35).

The AdaBoost model is distinguished by several key features. It leverages existing demographic, laboratory, and questionnaire data from the US NHANES, obviating the need for new data collection. This model integrates data from various sources to pinpoint the top 10 variables crucial for ML applications. Furthermore, between 2009 and 2013, heightened attention by the US government on heavy metal exposure, particularly in the context of environmental health, led to the initiation of numerous environmental governance initiatives (36). These initiatives resulted in reduced heavy metal levels and diverse MetS occurrences. Our ML models were developed and assessed using detailed participant data on blood heavy metal concentrations. Despite a general decline in heavy metal levels during 2009–2013, the NHANES dataset represents a single-time participation for individuals, implying that the heavy metal data does not reflect annual average exposure levels. However, this did not compromise the models’ stability, with the AdaBoost model demonstrating consistent reliability, indicated by an average AUC of 0.807. In addition to AdaBoost, we explored nine other ML techniques to detect MetS based on heavy metal exposure, drawing from recent cardiovascular disease research for enhanced insights. Certain models showed greater robustness and predictive accuracy with the incorporation of more granular data (37). An exhaustive evaluation of the ML models’ predictive capabilities revealed that the AdaBoost model stood out for its superior classification performance, underscored by nine distinguishing features.

This research presents novel insights, yet it is constrained by certain limitations that warrant acknowledgment. The cross-sectional nature of this study curtails the ability to establish causality or temporal sequences. Although the presence of heavy metals in biological specimens might indicate a connection with MetS or its constituents, the singular biomarker assessments in this study provide only a snapshot of exposure, with blood lead levels particularly reflecting recent exposures. Moreover, the dependence on self-reported data for MetS diagnosis in the US NHANES survey introduces a potential for information bias, including inaccuracies related to memory, which could affect the precision of the ML models in pinpointing MetS. The lack of an external validation group within our study design also limits the possibility of further substantiating the model’s reliability and its generalizability to a wider population.

Future research should emphasize the need for longitudinal studies to elucidate the causal connections between exposure to heavy metals and POPs and MetS. Exploring alternative biological matrices such as toenails or hair could provide more dependable indicators of long-term metal exposure, albeit with potential challenges related to measurement precision and varying growth rates. Subsequent inquiries should delve into the underlying mechanisms by which such exposures influence metabolic health, potentially through pathways like oxidative stress, inflammation, or hormonal disruption. A comprehensive understanding of the combined impacts of various metals and pollutants is crucial for developing effective prevention and intervention strategies. Expanding research to encompass diverse populations and settings will enhance the relevance and applicability of the findings, thereby informing public health initiatives tailored to the unique needs and risks of different communities. Furthermore, continuous analysis and interpretation of critical features will empower professionals with the insights necessary for informed decision-making, transcending mere dependency on algorithmic outputs. Efforts should also focus on validating the performance of models by enlarging the database and refining the interface between healthcare providers and ML models to improve their interpretability and practicality in clinical contexts.

## 5 Conclusion

In our study, the AdaBoost model exhibited exceptional effectiveness, precision, and resilience in identifying the association between heavy metal exposure and the incidence of MetS in participants from the US NHANES spanning 2003 to 2018. Employing transparent methods, we identified cadmium, molybdenum, cobalt, cesium, uranium, and barium as key contributors to the model’s predictive performance. Our results highlight the benefits of integrating machine learning approaches with SHAP techniques to explore the intricate impact of environmental exposures on health. Additionally, the predictive framework established by this research holds promise for devising targeted interventions to prevent and control MetS.

## Data availability statement

The original contributions presented in the study are included in the article/Supplementary material, further inquiries can be directed to the corresponding authors.

## Ethics statement

The ethics review board of the National Center for Health Statistics approved all NHANES protocols and written informed consents were obtained from all participants or their proxies (< 18 years). The study was performed in accordance with the ethical standards laid down in the 1964 Declaration of Helsinki and its later amendments. Bioethics Committee of Southern Medical University reviewed our study and have waived the need for ethical approval.

## Author contributions

JY: Writing – original draft. ZD: Investigation, Software, Writing – original draft. FY: Formal analysis, Methodology, Writing – original draft. RD: Writing – review & editing. TF: Writing – review & editing.

## References

[1] Hirode G Wong RJ. Trends in the prevalence of metabolic syndrome in the United States, 2011–2016. JAMA. (2020) 323:2526–8. doi: 10.1001/jama.2020.4501, PMID: 32573660 PMC7312413

[2] Kassi E Pervanidou P Kaltsas G Chrousos G. Metabolic syndrome: definitions and controversies. BMC Med. (2011) 9:48. doi: 10.1186/1741-7015-9-48, PMID: 21542944 PMC3115896

[3] Yao F Bo Y Zhao L Li Y Ju L Fang H. Prevalence and influencing factors of metabolic syndrome among adults in China from 2015 to 2017. Nutrients. (2021) 13:4475. doi: 10.3390/nu13124475, PMID: 34960027 PMC8705649

[4] Saklayen MG. The global epidemic of the metabolic syndrome. Curr Hypertens Rep. (2018) 20:12. doi: 10.1007/s11906-018-0812-z, PMID: 29480368 PMC5866840

[5] Lee BK Kim Y. Blood cadmium, mercury, and lead and metabolic syndrome in South Korea: 2005-2010 Korean National Health and nutrition examination survey. Am J Ind Med. (2013) 56:682–92. doi: 10.1002/ajim.22107, PMID: 22911659

[6] Rhee SY Hwang YC Woo JT Sinn DH Chin SO Chon S. Blood lead is significantly associated with metabolic syndrome in Korean adults: an analysis based on the Korea National Health and nutrition examination survey (KNHANES), 2008. Cardiovasc Diabetol. (2013) 9:9. doi: 10.1186/1475-2840-12-9, PMID: 23302150 PMC3849944

[7] Noor N Zong G Seely EW Weisskopf M James-Todd T. Urinary cadmium concentrations and metabolic syndrome in U.S. adults: the National Health and nutrition examination survey 2001-2014. Environ Int. (2018) 121:349–56. doi: 10.1016/j.envint.2018.08.029, PMID: 30243183 PMC6786759

[8] Rotter I Kosik-Bogacka D Dołęgowska B Safranow K Lubkowska A Laszczyńska M. Relationship between the concentrations of heavy metals and bioelements in aging men with metabolic syndrome. Int J Environ Res Public Health. (2015) 12:3944–61. doi: 10.3390/ijerph120403944, PMID: 25867198 PMC4410226

[9] Wang SL Chang FH Liou SH Wang HJ Li WF Hsieh DP. Inorganic arsenic exposure and its relation to metabolic syndrome in an industrial area of Taiwan. Environ Int. (2007) 33:805–11. doi: 10.1016/j.envint.2007.03.004, PMID: 17481731

[10] Rahman M Tondel M Ahmad SA Chowdhury IA Faruquee MH Axelson O. Hypertension and arsenic exposure in Bangladesh. Hypertension. (1999) 33:74–8. doi: 10.1161/01.hyp.33.1.74, PMID: 9931084

[11] Kuo CC Su PH Sun CW Liu HJ Chang CL Wang SL. Early-life arsenic exposure promotes atherogenic lipid metabolism in adolescence: a 15-year birth cohort follow-up study in Central Taiwan. Environ Int. (2018) 118:97–105. doi: 10.1016/j.envint.2018.05.033, PMID: 29859944

[12] Ngiam KY Khor IW. Big data and machine learning algorithms for health-care delivery. Lancet Oncol. (2019) 20:e262–73. doi: 10.1016/s1470-2045(19)30149-4, PMID: 31044724

[13] Dinh A Miertschin S Young A Mohanty SD. A data-driven approach to predicting diabetes and cardiovascular disease with machine learning. BMC Med Inform Decis Mak. (2019) 19:211. doi: 10.1186/s12911-019-0918-5, PMID: 31694707 PMC6836338

[14] Stafford IS Kellermann M Mossotto E Beattie RM Mac Arthur BD Ennis S. A systematic review of the applications of artificial intelligence and machine learning in autoimmune diseases. NPJ Digit Med. (2020) 3:30. doi: 10.1038/s41746-020-0229-3, PMID: 32195365 PMC7062883

[15] Alber M Buganza Tepole A Cannon WR De S Dura-Bernal S Garikipati K. Integrating machine learning and multiscale modeling-perspectives, challenges, and opportunities in the biological, biomedical, and behavioral sciences. NPJ Digit Med. (2019) 2:115. doi: 10.1038/s41746-019-0193-y, PMID: 31799423 PMC6877584

[16] McQuillan GM McLean JE Chiappa M Corporation H Lukacs SL. National Health and nutrition examination survey biospecimen program: NHANES III (1988-1994) and NHANES 1999-2014. Vital Health Stat 2. (2015):1–14. PMID: 26222898

[17] American Diabetes Association. Economic costs of diabetes in the U.S. in 2017. Diabetes Care. (2018) 2018, 41:917–28. doi: 10.2337/dci18-0007, PMID: 29567642 PMC5911784

[18] Alberti KG Eckel RH Grundy SM Zimmet PZ Cleeman JI Donato KA. Harmonizing the metabolic syndrome: a joint interim statement of the international diabetes federation task force on epidemiology and prevention; National Heart, Lung, and Blood Institute; American Heart Association; world heart federation; international atherosclerosis society; and International Association for the Study of obesity. Circulation. (2009) 120:1640–5. doi: 10.1161/circulationaha.109.192644, PMID: 19805654

[19] Pruessner JC Kirschbaum C Meinlschmid G Hellhammer DH. Two formulas for computation of the area under the curve represent measures of total hormone concentration versus time-dependent change. Psychoneuroendocrinology. (2003) 28:916–31. doi: 10.1016/s0306-4530(02)00108-7, PMID: 12892658

[20] Rudin C. Stop explaining black box machine learning models for high stakes decisions and use interpretable models instead. Nat Mach Intell. (2019) 1:206–15. doi: 10.1038/s42256-019-0048-x, PMID: 35603010 PMC9122117

[21] Akyea RK Qureshi N Kai J Weng SF. Performance and clinical utility of supervised machine-learning approaches in detecting familial hypercholesterolaemia in primary care. NPJ Digit Med. (2020) 3:142. doi: 10.1038/s41746-020-00349-5, PMID: 33145438 PMC7603302

[22] Weng SF Reps J Kai J Garibaldi JM Qureshi N. Can machine-learning improve cardiovascular risk prediction using routine clinical data? PLoS One. (2017) 12:e0174944. doi: 10.1371/journal.pone.0174944, PMID: 28376093 PMC5380334

[23] Sufriyana H Husnayain A Chen YL Kuo CY Singh O Yeh TY. Comparison of multivariable logistic regression and other machine learning algorithms for prognostic prediction studies in pregnancy care: systematic review and Meta-analysis. JMIR Med Inform. (2020) 8:e16503. doi: 10.2196/16503, PMID: 33200995 PMC7708089

[24] Mac Eachern SJ Forkert ND. Machine learning for precision medicine. Genome. (2021) 64:416–25. doi: 10.1139/gen-2020-0131, PMID: 33091314

[25] Huang Y He C Shen C Guo J Mubeen S Yuan J. Toxicity of cadmium and its health risks from leafy vegetable consumption. Food Funct. (2017) 8:1373–401. doi: 10.1039/c6fo01580h, PMID: 28232985

[26] Giannico OV Fragnelli GR Baldacci S Desiante F Pellegrino A Basile FC. Dioxins and PCBs contamination in milk and dairy products from province of Taranto (Puglia region, southern Italy): a six years spatio-temporal monitoring study. Ann Ist Super Sanita. (2021) 57:233–8. doi: 10.4415/ann_21_03_06, PMID: 34554117

[27] Giannico OV Baldacci S Basile FC Pellegrino A Desiante F Franco E. PCDD/fs and PCBs in hen eggs from a contaminated area in Italy: a 9 years spatio-temporal monitoring study. Food Addit Contam Part A Chem Anal Control Expo Risk Assess. (2023) 40:294–304. doi: 10.1080/19440049.2022.2157051, PMID: 36602427

[28] Giannico OV Baldacci S Desiante F Basile FC Franco E Fragnelli GR. PCDD/fs and PCBs in *Mytilus galloprovincialis* from a contaminated area in Italy: the role of mussel size, temperature and meteorological factors. Food Addit Contam Part A Chem Anal Control Expo Risk Assess. (2022) 39:1123–35. doi: 10.1080/19440049.2022.2059108, PMID: 35389328

[29] Lee BK Kim Y. Association of Blood Cadmium Level with metabolic syndrome after adjustment for confounding by serum ferritin and other factors: 2008-2012 Korean National Health and nutrition examination survey. Biol Trace Elem Res. (2016) 171:6–16. doi: 10.1007/s12011-015-0499-9, PMID: 26343361

[30] Ghaedrahmat Z Cheraghian B Jaafarzadeh N Takdastan A Shahbazian HB Ahmadi M. Relationship between urinary heavy metals with metabolic syndrome and its components in population from Hoveyzeh cohort study: a case-control study in Iran. J Trace Elem Med Biol. (2021) 66:126757. doi: 10.1016/j.jtemb.2021.126757, PMID: 33839459

[31] Flores CR Puga MP Wrobel K Garay Sevilla ME Wrobel K. Trace elements status in diabetes mellitus type 2: possible role of the interaction between molybdenum and copper in the progress of typical complications. Diabetes Res Clin Pract. (2011) 91:333–41. doi: 10.1016/j.diabres.2010.12.014, PMID: 21211861

[32] Valko M Morris H Cronin MT. Metals, toxicity and oxidative stress. Curr Med Chem. (2005) 12:1161–208. doi: 10.2174/0929867053764635, PMID: 15892631

[33] Xu P Liu A Li F Tinkov AA Liu L Zhou JC. Associations between metabolic syndrome and four heavy metals: a systematic review and meta-analysis. Environ Pollut. (2021) 15:116480. doi: 10.1016/j.envpol.2021.116480, PMID: 33486246

[34] Vahter M Concha G. Role of metabolism in arsenic toxicity. Pharmacol Toxicol. (2001) 89:1–5. doi: 10.1034/j.1600-0773.2001.d01-128.x, PMID: 11484904

[35] Tchounwou PB Yedjou CG Patlolla AK Sutton DJ. Heavy metal toxicity and the environment. Exp Suppl. (2012) 101:133–64. doi: 10.1007/978-3-7643-8340-4_6, PMID: 22945569 PMC4144270

[36] Southon A Burke R Camakaris J. What can flies tell us about copper homeostasis? Metallomics. (2013) 5:1346–56. doi: 10.1039/c3mt00105a, PMID: 23903872

[37] Kunze KN Polce EM Patel A Courtney PM Sporer SM Levine BR. Machine learning algorithms predict within one size of the final implant ultimately used in total knee arthroplasty with good-to-excellent accuracy. Knee Surg Sports Traumatol Arthrosc. (2022) 30:2565–72. doi: 10.1007/s00167-022-06866-y, PMID: 35024899

