# Corrigendum: The relationship between heavy metals and metabolic syndrome using machine learning

**DOI:** 10.3389/fpubh.2024.1456139

**Published:** 2024-07-29

**Authors:** Jun Yao, Zhilin Du, Fuyue Yang, Ran Duan, Tong Feng

**Affiliations:** ^1^Department of Respiratory and Critical Care, Guangyuan Central Hospital, Guangyuan, Sichuan, China; ^2^Department of Oncology, Chengdu Seventh People's Hospital (Affliated Cancer Hospital of Chengdu Medical College), Chengdu, Sichuan, China; ^3^Department of Rheumatology and Immunology, Chengdu Fifth People's Hospital, Chengdu, Sichuan, China; ^4^Clinical Medical College, Chengdu Medical College, Chengdu, Sichuan, China; ^5^Department of Oncology, The First Affiliated Hospital of Chengdu Medical College, Chengdu, Sichuan, China; ^6^The Second School of Clinical Medicine, Southern Medical University, Guangzhou, China

**Keywords:** metabolic syndrome, NHANES (National Health and Nutrition Examination Survey), machine learning, heavy metals, SHapley additive exPlanations (SHAP)

In the published article, there was an error regarding the affiliations for Ran Duan. In addition to being affiliated with “Department of Oncology, The First Affiliated Hospital of Chengdu Medical College, Chengdu, Sichuan, China,” she should also be affiliated with “Clinical Medical College, Chengdu Medical College, Chengdu, Sichuan, China.”

The authors apologize for this error and state that this does not change the scientific conclusions of the article in any way. The original article has been updated.

