# Supplementary material for: The relationship between heavy metals and metabolic syndrome using machine learning
Source: Front Public Health. 2024 Apr 15;12:1378041. doi: 10.3389/fpubh.2024.1378041 (PMC11057329; doi:10.3389/fpubh.2024.1378041)
Supplement: Supplementary file 1 [file Data_Sheet_1.doc]

Title “Predicting Metabolic Syndrome Using Machine Learning: The Role of Heavy Metal Exposure”

**Supplementary Materials**

**Supplementary Table Legend**

**Supplementary Table 1.** Description of missing category variables

**Supplementary Table 2.** Description of missing quantitative variables

**Supplementary Table 3.**Distribution of heavy metal data before outlier handling

**Supplementary Table 4.**Distribution of heavy metal data after outlier handling

**Supplementary Table 5.**Distribution of heavy metal data after natural logarithm conversion

**Supplementary Table 6.** Details of hyperparameter for ML models.

**Supplementary Table7.** Assessment of multicollinearity among model variables using variance inflation factors (VIF).

**Supplementary Table 8.** AUC Delong test of nine machine learning models

**Supplementary Figure Legend**

**Supplementary Figure 1.** Flow Chart of the Study

**Supplementary Figure 2.** Distribution of variables before and after interpolation

**Supplementary Figure 3.** The results of Pearson's correlation analysis among the 18 metal variables.

**Supplementary Figure 4.** Confusion Matrix of Models

**Supplementary material.**

**Predictive Model**

Machine learning is a subfield of artificial intelligence that focuses on the development of algorithms and models to learn from data, automatically improve performance, and make predictions. It enables computers to recognize patterns, understand complex data, and make informed decisions without being explicitly programmed. In the context of machine learning, classification refers to the process of assigning labeled outputs to unlabeled inputs based on their characteristics.

1. XGBClassifier: XGBoost (Extreme Gradient Boosting) is a powerful machine learning algorithm known for its high accuracy and speed. It is an ensemble learning method that combines multiple weak classifiers (decision trees) to create a strong classifier.

2. LogisticRegression: Logistic regression is a simple, yet powerful classification algorithm. It uses a sigmoid function to convert the linear combination of input features into binary outputs (0 or 1) for binary classification problems.

3. RandomForestClassifier: Random forest is another ensemble learning method that combines multiple decision trees to create a strong classifier. It randomly selects a subset of features and samples from the training data to build each tree, resulting in a robust and efficient model.

4. AdaBoostClassifier: AdaBoost (Adaptive Boosting) is a meta-algorithm that combines multiple weak classifiers (such as decision trees) to create a strong classifier. It works by adjusting the weights of the training samples and combining the predictions of the weak classifiers to produce a final prediction.

5. GaussianNB: Gaussian naive Bayes (GNB) is a probabilistic classification algorithm that assumes the input features follow a Gaussian distribution. It is particularly useful for problems with continuous features and relies on the Bayes' rule to calculate the probability of each class.

6. ComplementNB: Complement naive Bayes (CNB) is a variation of GNB that uses the complement rule to handle class imbalance and improve prediction performance.

7. MLPClassifier: Multilayer perceptron (MLP) is a type of neural network that consists of multiple layers of neurons. It is a powerful machine learning algorithm for classification and regression tasks, but can be computationally expensive and prone to overfitting.

8. SVC: Support vector classifier (SVC) is a popular kernel-based classification algorithm that uses a hyperplane to separate the data into different classes. It is particularly useful for high-dimensional data and can handle both linear and non-linear problems.

1. KNeighborsClassifier: k-Nearest neighbors (KNN) is a simple yet effective classification algorithm. It assigns labels to new inputs based on their proximity to training samples of known labels, using the majority vote or other distance-based methods.

**Interpretable methods of model**

SHAP values provide a global explanation for the predictions of any machine learning model, including complex neural networks. They are based on game theory and cooperative game theory, specifically the Shapley value concept. SHAP values assign a contribution score to each feature, representing its impact on the prediction.

**Supplementary Table 1.** Description of missing category variables

| variable | total | taxonomy term | Highest frequency item | frequency | Missing rate% |
| --- | --- | --- | --- | --- | --- |
| MetS | 11667 | 2 | 0 | 7827 | 0.000 |
| alcoholuser | 11667 | 6 | 3.0 | 3400 | 8.305 |
| smoke | 11667 | 4 | 0.0 | 6366 | 0.026 |
| edu | 11667 | 4 | 3.0 | 5896 | 0.060 |
| eth | 11667 | 5 | 3 | 5188 | 0.000 |
| sex | 11667 | 2 | 1 | 5951 | 0.000 |

**Supplementary Table 2.** Description of missing quantitative variables

| variable | total | mean | median | 25% quantile | 75% quantile | standard deviation | minimum value | maximum value | missing rate% |
| --- | --- | --- | --- | --- | --- | --- | --- | --- | --- |
| molybdenum | 11667.000 | 54.572 | 41.110 | 21.800 | 70.600 | 53.297 | 0.650 | 1215.900 | 0.000 |
| uranium | 11667.000 | 0.012 | 0.006 | 0.003 | 0.012 | 0.033 | 0.001 | 1.824 | 0.000 |
| tungsten | 11667.000 | 0.126 | 0.068 | 0.031 | 0.133 | 0.395 | 0.010 | 32.910 | 0.000 |
| thallium | 11667.000 | 0.185 | 0.156 | 0.091 | 0.244 | 0.154 | 0.011 | 7.658 | 0.000 |
| antimony | 11667.000 | 0.088 | 0.051 | 0.029 | 0.093 | 0.226 | 0.016 | 14.291 | 0.000 |
| lead | 11667.000 | 0.740 | 0.490 | 0.260 | 0.880 | 1.257 | 0.020 | 52.300 | 0.000 |
| cesium | 11667.000 | 5.327 | 4.506 | 2.742 | 6.771 | 7.253 | 0.061 | 552.120 | 0.000 |
| cobalt | 11667.000 | 0.536 | 0.352 | 0.212 | 0.562 | 1.621 | 0.016 | 127.870 | 0.000 |
| Cadmium | 11667.000 | 0.388 | 0.243 | 0.120 | 0.480 | 0.462 | 0.025 | 6.940 | 0.000 |
| Barium | 11667.000 | 1.974 | 1.180 | 0.590 | 2.290 | 3.403 | 0.042 | 101.000 | 0.000 |
| dimethylarsonicacid | 11667.000 | 5.609 | 3.660 | 2.035 | 6.355 | 7.906 | 1.200 | 270.000 | 0.000 |
| Arsenocholine | 11667.000 | 0.348 | 0.400 | 0.160 | 0.420 | 1.219 | 0.080 | 107.000 | 0.000 |
| Arsenobetaine | 11667.000 | 10.610 | 1.280 | 0.820 | 6.175 | 42.108 | 0.280 | 1280.000 | 0.000 |
| Arsenicacid | 11667.000 | 0.697 | 0.700 | 0.560 | 0.710 | 1.463 | 0.560 | 148.000 | 0.000 |
| Arsenousacid | 11667.000 | 0.725 | 0.850 | 0.370 | 0.850 | 1.654 | 0.080 | 156.000 | 0.000 |
| Blood mercury | 11667.000 | 1.584 | 0.870 | 0.460 | 1.700 | 2.474 | 0.100 | 50.810 | 0.000 |
| Blood lead | 11667.000 | 1.658 | 1.250 | 0.790 | 2.000 | 1.631 | 0.050 | 33.670 | 0.000 |
| Blood cadmium | 11667.000 | 0.517 | 0.330 | 0.200 | 0.600 | 0.573 | 0.070 | 8.800 | 0.000 |
| BMI | 11535.000 | 28.936 | 27.800 | 24.265 | 32.300 | 6.756 | 13.180 | 82.900 | 1.131 |
| poverty | 10780.000 | 2.514 | 2.110 | 1.120 | 4.000 | 1.612 | 0.000 | 5.000 | 7.603 |
| age | 11667.000 | 49.361 | 48.000 | 34.000 | 64.000 | 18.008 | 20.000 | 85.000 | 0.000 |

**Supplementary Table 3.**Distribution of heavy metal data before outlier handling

| variable | total | mean | median | 25% quantile | 75% quantile | standard deviation | minimum value | maximum value |
| --- | --- | --- | --- | --- | --- | --- | --- | --- |
| molybdenum | 11667 | 54.572 | 41.11 | 21.8 | 70.6 | 0.65 | 1215.9 | 11667 |
| uranium | 11667 | 0.012 | 0.006 | 0.003 | 0.012 | 0.001 | 1.824 | 11667 |
| tungsten | 11667 | 0.126 | 0.068 | 0.031 | 0.133 | 0.01 | 32.91 | 11667 |
| thallium | 11667 | 0.185 | 0.156 | 0.091 | 0.244 | 0.011 | 7.658 | 11667 |
| antimony | 11667 | 0.088 | 0.051 | 0.029 | 0.093 | 0.016 | 14.291 | 11667 |
| lead | 11667 | 0.74 | 0.49 | 0.26 | 0.88 | 0.02 | 52.3 | 11667 |
| cesium | 11667 | 5.327 | 4.506 | 2.742 | 6.771 | 0.061 | 552.12 | 11667 |
| cobalt | 11667 | 0.536 | 0.352 | 0.212 | 0.562 | 0.016 | 127.87 | 11667 |
| Cadmium1 | 11667 | 0.388 | 0.243 | 0.12 | 0.48 | 0.025 | 6.94 | 11667 |
| Barium | 11667 | 1.974 | 1.18 | 0.59 | 2.29 | 0.042 | 101 | 11667 |
| dimethylarsonicacid | 11667 | 5.609 | 3.66 | 2.035 | 6.355 | 1.2 | 270 | 11667 |
| Arsenocholine | 11667 | 0.348 | 0.4 | 0.16 | 0.42 | 0.08 | 107 | 11667 |
| Arsenobetaine | 11667 | 10.61 | 1.28 | 0.82 | 6.175 | 0.28 | 1280 | 11667 |
| Arsenicacid | 11667 | 0.697 | 0.7 | 0.56 | 0.71 | 0.56 | 148 | 11667 |
| Arsenousacid | 11667 | 0.725 | 0.85 | 0.37 | 0.85 | 0.08 | 156 | 11667 |
| Blood mercury total | 11667 | 1.584 | 0.87 | 0.46 | 1.7 | 0.1 | 50.81 | 11667 |
| Blood lead | 11667 | 1.658 | 1.25 | 0.79 | 2 | 0.05 | 33.67 | 11667 |
| Blood cadmium | 11667 | 0.517 | 0.33 | 0.2 | 0.6 | 0.07 | 8.8 | 11667 |

**Supplementary Table 4.**Distribution of heavy metal data after outlier handling

| variable | total | mean | median | 25% quantile | 75% quantile | standard deviation | minimum value | maximum value |
| --- | --- | --- | --- | --- | --- | --- | --- | --- |
| molybdenum | 11667 | 45.951 | 41.11 | 21.8 | 63.4 | 0.65 | 143.75 | 11667 |
| uranium | 11667 | 0.007 | 0.006 | 0.003 | 0.009 | 0.001 | 0.024 | 11667 |
| tungsten | 11667 | 0.078 | 0.068 | 0.031 | 0.106 | 0.01 | 0.286 | 11667 |
| thallium | 11667 | 0.169 | 0.156 | 0.091 | 0.23 | 0.011 | 0.473 | 11667 |
| antimony | 11667 | 0.06 | 0.051 | 0.029 | 0.079 | 0.016 | 0.189 | 11667 |
| lead | 11667 | 0.553 | 0.49 | 0.26 | 0.74 | 0.02 | 1.81 | 11667 |
| cesium | 11667 | 4.806 | 4.506 | 2.742 | 6.45 | 0.061 | 12.8 | 11667 |
| cobalt | 11667 | 0.374 | 0.352 | 0.212 | 0.49 | 0.016 | 1.081 | 11667 |
| Cadmium1 | 11667 | 0.288 | 0.243 | 0.12 | 0.392 | 0.025 | 1.02 | 11667 |
| Barium | 11667 | 1.385 | 1.18 | 0.59 | 1.885 | 0.042 | 4.84 | 11667 |
| dimethylarsonicacid | 11667 | 4.055 | 3.66 | 2.035 | 5.23 | 1.2 | 12.8 | 11667 |
| Arsenocholine | 11667 | 0.302 | 0.4 | 0.16 | 0.42 | 0.08 | 0.81 | 11667 |
| Arsenobetaine | 11667 | 2.272 | 1.28 | 0.82 | 2.49 | 0.28 | 14.2 | 11667 |
| Arsenicacid | 11667 | 0.653 | 0.7 | 0.56 | 0.71 | 0.56 | 0.93 | 11667 |
| Arsenousacid | 11667 | 0.668 | 0.85 | 0.37 | 0.85 | 0.08 | 1.56 | 11667 |
| Blood mercury total | 11667 | 0.997 | 0.87 | 0.46 | 1.295 | 0.1 | 3.55 | 11667 |
| Blood lead | 11667 | 1.369 | 1.25 | 0.79 | 1.78 | 0.05 | 3.81 | 11667 |
| Blood cadmium | 11667 | 0.381 | 0.33 | 0.2 | 0.48 | 0.07 | 1.2 | 11667 |

**Supplementary Table 5.**Distribution of heavy metal data after natural logarithm conversion

| variable | total | mean | median | 25% quantile | 75% quantile | minimum value | Maximum value |
| --- | --- | --- | --- | --- | --- | --- | --- |
| blood_cadmium | 11667 | -1.158 | -1.109 | -1.609 | -0.734 | -2.659 | 0.182 |
| blood_lead | 11667 | 0.15 | 0.223 | -0.236 | 0.577 | -2.996 | 1.338 |
| blood_mercury_total | 11667 | -0.272 | -0.139 | -0.777 | 0.259 | -2.303 | 1.267 |
| Arsenousacid | 11667 | -0.613 | -0.163 | -0.994 | -0.163 | -2.526 | 0.445 |
| Arsenicacid | 11667 | -0.431 | -0.357 | -0.58 | -0.342 | -0.58 | -0.073 |
| Arsenobetaine | 11667 | 0.233 | 0.247 | -0.198 | 0.912 | -1.273 | 2.653 |
| Arsenocholine | 11667 | -1.392 | -0.916 | -1.833 | -0.868 | -2.526 | -0.211 |
| dimethylarsonicacid | 11667 | 1.201 | 1.297 | 0.71 | 1.654 | 0.182 | 2.549 |
| Barium | 11667 | 0.014 | 0.166 | -0.528 | 0.634 | -3.17 | 1.577 |
| Cadmium | 11667 | -1.575 | -1.415 | -2.12 | -0.936 | -3.689 | 0.02 |
| cobalt | 11667 | -1.17 | -1.044 | -1.551 | -0.713 | -4.135 | 0.078 |
| cesium | 11667 | 1.391 | 1.505 | 1.009 | 1.864 | -2.797 | 2.549 |
| lead | 11667 | -0.855 | -0.713 | -1.347 | -0.301 | -3.912 | 0.593 |
| antimony | 11667 | -2.997 | -2.976 | -3.54 | -2.538 | -4.135 | -1.666 |
| thallium | 11667 | -1.977 | -1.858 | -2.397 | -1.47 | -4.51 | -0.749 |
| tungsten | 11667 | -2.852 | -2.688 | -3.474 | -2.244 | -4.605 | -1.252 |
| uranium | 11667 | -5.252 | -5.15 | -5.714 | -4.722 | -6.908 | -3.742 |
| molybdenum | 11667 | 3.561 | 3.716 | 3.082 | 4.149 | -0.431 | 4.968 |

**Supplementary Table 6.** Details of hyperparameter for ML models.

| Model | Hyperparameter |
| --- | --- |
| XGB Classifier | Reg_ Lambda (L2 regularization coefficient): 1 min_ Child_ Weight (minimum fork weight sum): 6 max_ Depth (maximum tree depth): 4 learning_ Rate: 0.1 |
| Logistic Regression | Tol (convergence metric): 0.0001 penalty (regularization type): l2 max_ Iter (number of iterations): 100 C (regularization factor): 9.9999999999999999e-05 |
| RF  Classifier | N_ Estimators (number of trees): 100 minutes_ Impurity_ Decrease (minimum bifurcation purity gain): 0.0 max_ Depth: None criterion: gini |
| AB  Classifier | N_ Estimators (number of single models): 50 learning_ Rate: 0.1 |
| Gaussian  NB | var_smoothing：1e-07 |
| Complement  NB | Alpha (Laplace/Lidstone smoothing): 0 |
| MLP  Classifier | Max_ Iter (number of iterations): 10 hidden_ Layer_ Sizes (hidden layer width): (10, 10) activation (non-linear function): logistic |
| SVC | Tol (convergence metric): 0.001 kernel (kernel type): rbf C (regularization factor): 0.1 |
| KNN  Classifier | Weights (weight type): uniform n_ Neighbors: 2 |

ML, machine learning. XGB,extreme gradient boosting.RF, random forest. SVC, support vector classification. MLP, multilayer perceptron. AB, AdaBoost. KNN, K-Nearest Neighbor.NB, Naive Bayes.

**Supplementary Table 7.**Assessment of multicollinearity among model variables using variance inflation factors (VIF).

| Feature | VIF |
| --- | --- |
| cesium1 | 3.394 |
| lead1 | 3.114 |
| thallium1 | 2.826 |
| cobalt1 | 2.227 |
| molybdenum1 | 2.191 |
| Cadmium1 | 2.124 |
| Arsenousacid | 2.080 |
| Arsenicacid | 2.068 |
| blood_lead_ugdl | 2.052 |
| dimethylarsonicacid | 1.832 |
| age | 1.693 |
| antimony1 | 1.658 |
| tungsten1 | 1.652 |
| blood_cadmium_ugl | 1.550 |
| Barium1 | 1.466 |
| uranium1 | 1.450 |
| smoke | 1.430 |
| poverty | 1.285 |
| edu | 1.284 |
| alcoholuser | 1.238 |
| blood_mercury_total_ugl | 1.236 |
| Arsenobetaine | 1.236 |
| BMI_kgm2 | 1.065 |

**Supplementary Table 8.** AUC Delong test of nine machine learning models

| name | XGBClassifier | LogisticRegression | RandomForestClassifier | AdaBoostClassifier | GaussianNB | ComplementNB | MLPClassifier | SVC | KNeighborsClassifier |
| --- | --- | --- | --- | --- | --- | --- | --- | --- | --- |
| XGBClassifier | NA | 0.0 | 0.0 | 0.0 | 0.0 | 0.0 | 0.0 | 0.0 | 0.0 |
| LogisticRegression | 0.0 | NA | 0.61 | 0.835 | 0.526 | 0.835 | 0.706 | 0.42 | 0.716 |
| RandomForestClassifier | 0.0 | 0.61 | NA | 0.467 | 0.456 | 0.749 | 0.405 | 0.771 | 0.887 |
| AdaBoostClassifier | 0.0 | 0.835 | 0.467 | NA | 0.566 | 0.691 | 0.758 | 0.309 | 0.559 |
| GaussianNB | 0.0 | 0.526 | 0.456 | 0.566 | NA | 0.631 | 0.789 | 0.324 | 0.52 |
| ComplementNB | 0.0 | 0.835 | 0.749 | 0.691 | 0.631 | NA | 0.594 | 0.542 | 0.862 |
| MLPClassifier | 0.0 | 0.706 | 0.405 | 0.758 | 0.789 | 0.594 | NA | 0.268 | 0.488 |
| SVC | 0.0 | 0.42 | 0.771 | 0.309 | 0.324 | 0.542 | 0.268 | NA | 0.661 |
| KNeighborsClassifier | 0.0 | 0.716 | 0.887 | 0.559 | 0.52 | 0.862 | 0.488 | 0.661 | NA |

**
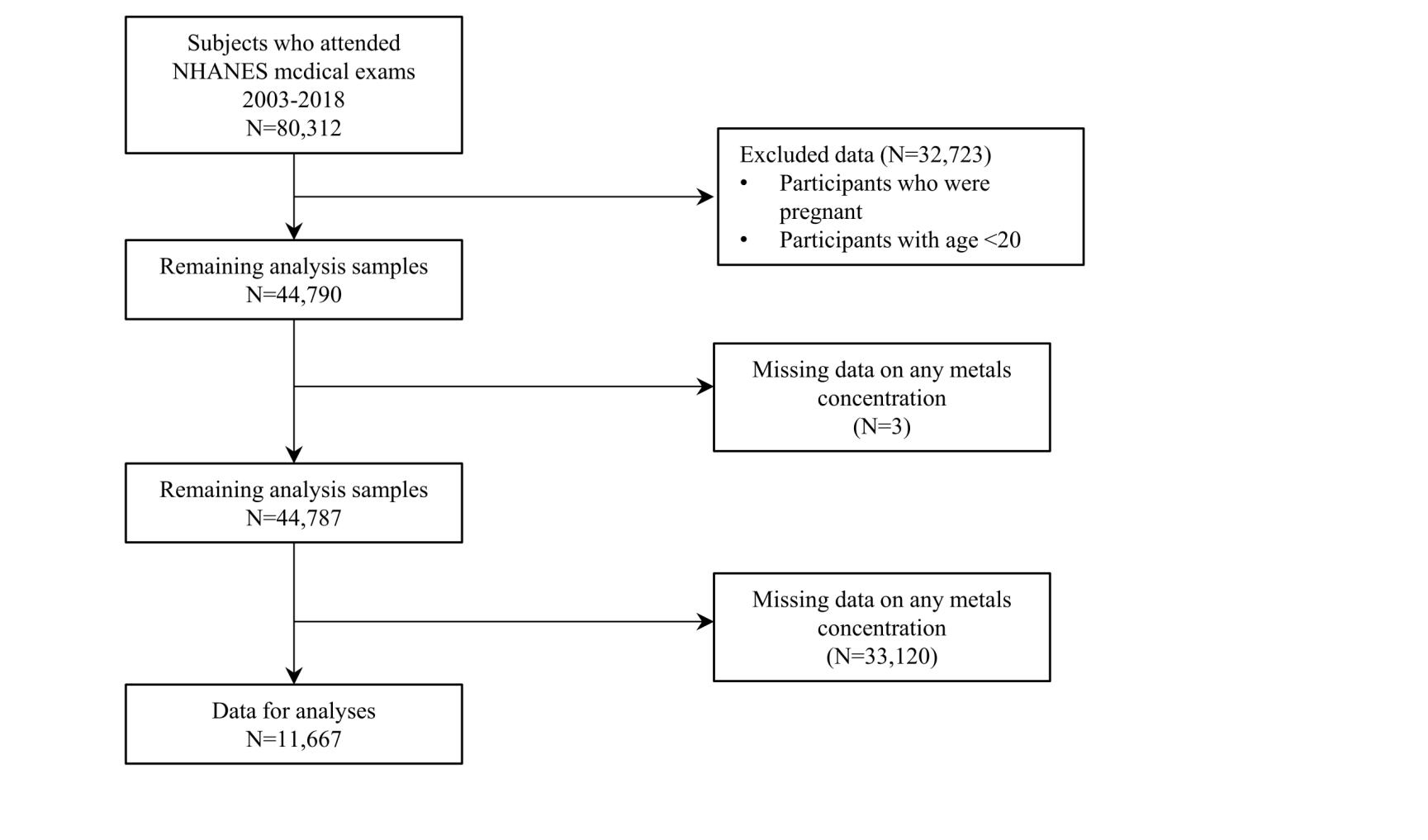
**

**Supplemental Figure 1.** Flowchart for the selection of eligible participants.


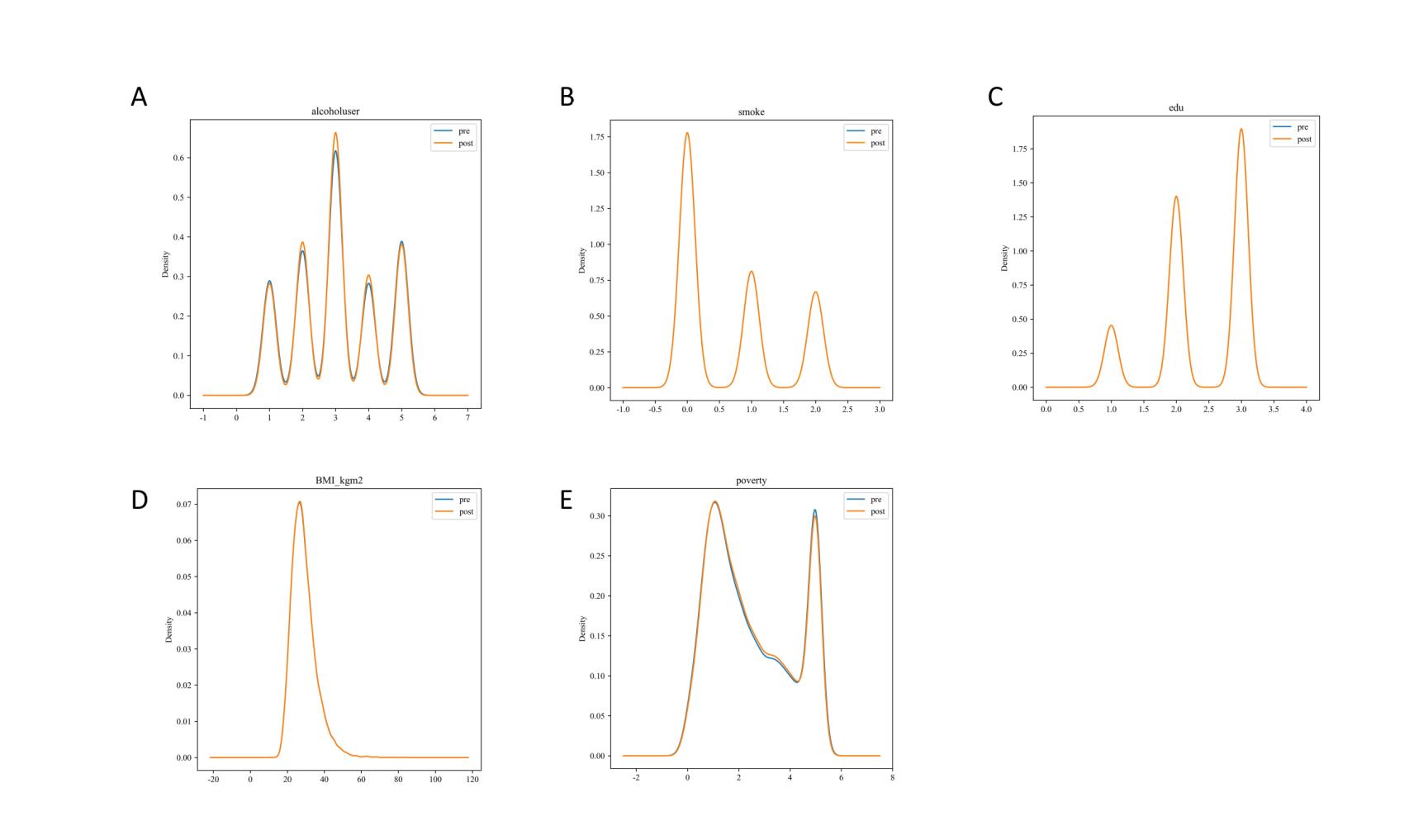


**Supplemental Figure 2.** Distribution of variables before and after interpolation

**
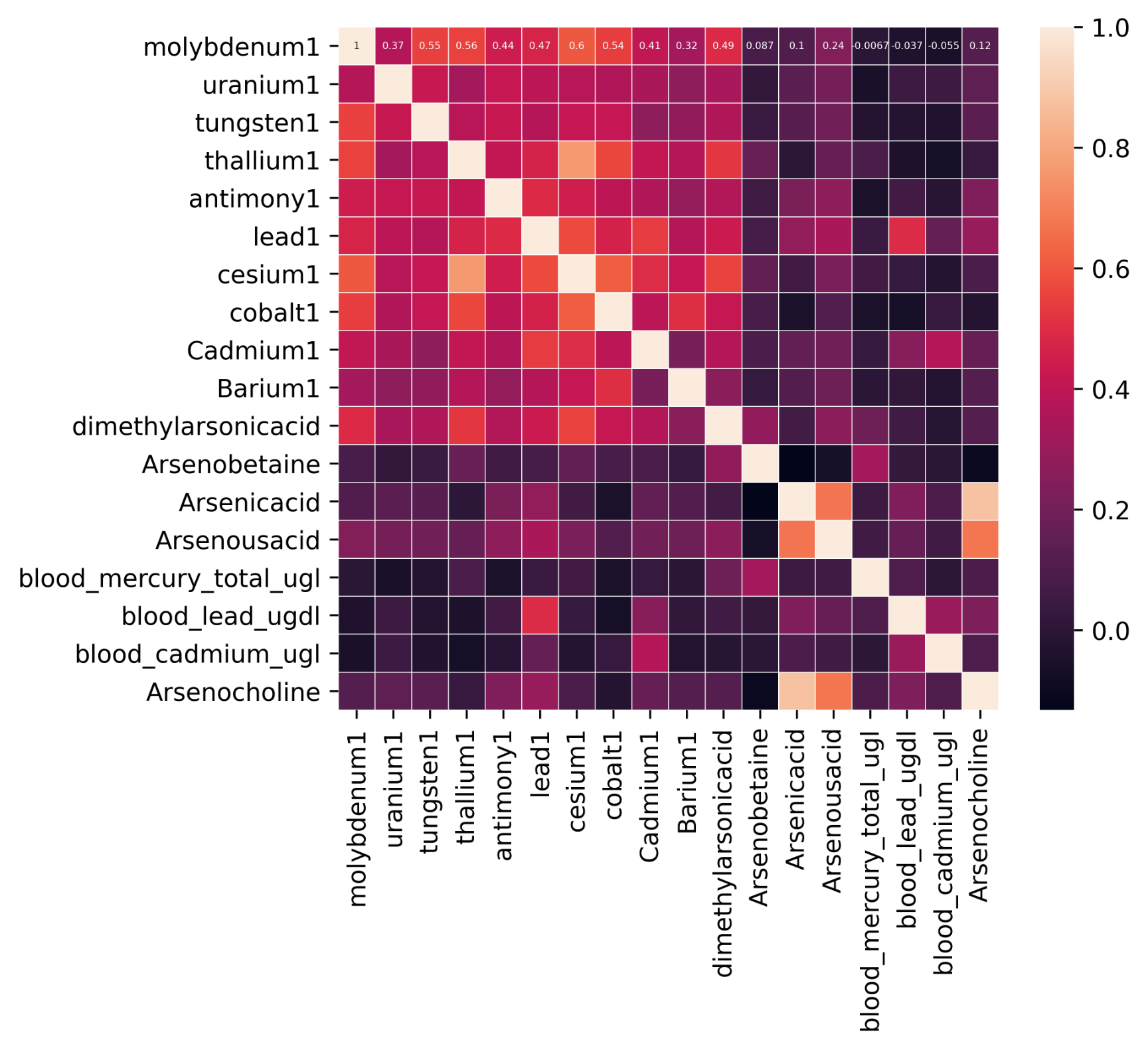
**

**Supplemental Figure 3.** The results of Pearson's correlation analysis among the 18 metal variables.


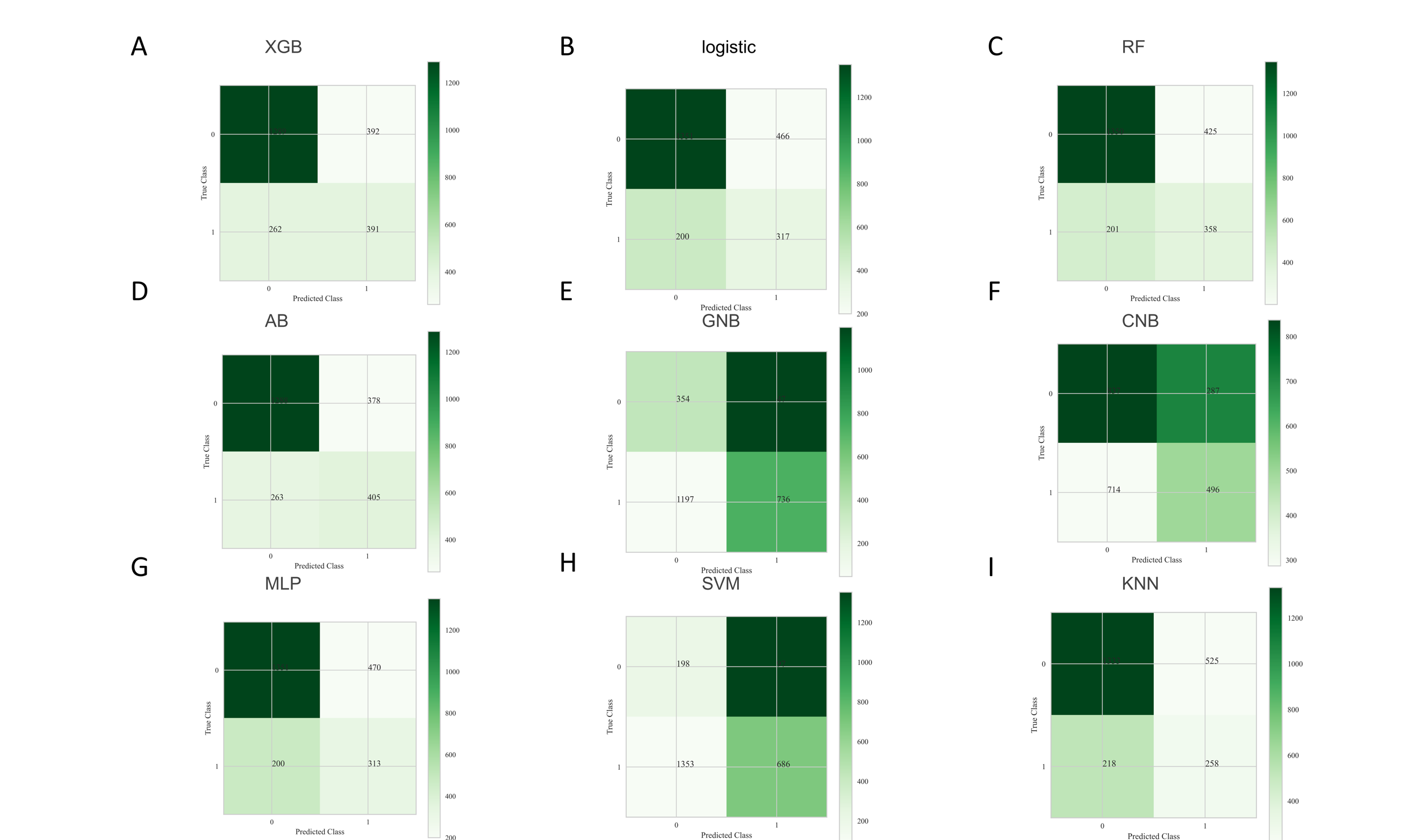


**Supplemental Figure 4.** The confusion matrix of the nine ML model
